# Partitioning into ER membrane microdomains impacts autophagic protein turnover during cellular aging

**DOI:** 10.1038/s41598-024-64493-8

**Published:** 2024-06-13

**Authors:** Simon Prokisch, Sabrina Büttner

**Affiliations:** Department of Molecular Biosciences, The Wenner-Gren Institute, Stockholm University, 10691 Stockholm, Sweden

**Keywords:** Endoplasmic reticulum, Autophagy, Cellular imaging, Endoplasmic reticulum

## Abstract

Eukaryotic membranes are compartmentalized into distinct micro- and nanodomains that rearrange dynamically in response to external and internal cues. This lateral heterogeneity of the lipid bilayer and associated clustering of distinct membrane proteins contribute to the spatial organization of numerous cellular processes. Here, we show that membrane microdomains within the endoplasmic reticulum (ER) of yeast cells are reorganized during metabolic reprogramming and aging. Using biosensors with varying transmembrane domain length to map lipid bilayer thickness, we demonstrate that in young cells, microdomains of increased thickness mainly exist within the nuclear ER, while progressing cellular age drives the formation of numerous microdomains specifically in the cortical ER. Partitioning of biosensors with long transmembrane domains into these microdomains increased protein stability and prevented autophagic removal. In contrast, reporters with short transmembrane domains progressively accumulated at the membrane contact site between the nuclear ER and the vacuole, the so-called nucleus-vacuole junction (NVJ), and were subjected to turnover via selective microautophagy occurring specifically at these sites. Reporters with long transmembrane domains were excluded from the NVJ. Our data reveal age-dependent rearrangement of the lateral organization of the ER and establish transmembrane domain length as a determinant of membrane contact site localization and autophagic degradation.

## Introduction

Cellular compartmentalization into membrane-delimited organelles allows the spatial separation of various, often incompatible, biochemical reactions. Each intracellular membrane possesses its own characteristic lipid profile, thereby specifying organelle identity and biophysical membrane properties. Further subcompartmentalization and organization within each lipid bilayer at the micro- and nanoscale is achieved by the formation of lateral membrane subdomains, among them lipid rafts^1–4^. Following the initial proposal of the lipid raft hypothesis^4^, the definition of lipid rafts has been refined to "small (10–200 nm), heterogeneous, highly dynamic, sterol- and sphingolipid-enriched domains that compartmentalize cellular processes. Small rafts can sometimes be stabilized to form larger platforms through protein–protein and protein-lipid interactions" almost 20 years ago^5^. Until today, controversies exist in respect to their life time, size, composition and properties in biological membranes. These membrane subdomains have been shown to represent versatile platforms for the trafficking and sorting of associated proteins^4–7^. The lateral heterogeneity in biological membranes is studied intensively in particular in respect to lipid rafts associated with the plasma membrane^8^. Still, raft-like microdomains also exist in organellar lipid bilayers, including the membranes of the endoplasmic reticulum (ER), despite the sterol and sphingolipid content within the ER membrane being rather low compared to the plasma membrane^1,8–10^. The ER represents the largest intracellular membrane system and serves as entry point into the secretory pathway, giving rise to almost 30% of the proteome of eukaryotic cells, mostly membrane proteins^11–14^. These membrane proteins carry one or more transmembrane domains (TMDs), composed of 17–29 hydrophobic amino acids that span the lipid bilayer as alpha-helices^14–16^. The sorting of these membrane proteins to their final compartment within the secretory pathway is strongly influenced by physiochemical features of their TMDs, including length, hydrophobicity and amino acid volume^17–19^. The mean TMD length is 20.6 amino acids for membrane proteins retained within the ER, 23.6 amino acids for those in the trans-Golgi network and 27.0 for proteins in the plasma membrane^20^, reflecting the need to match the increasing thickness of the lipid bilayer along the secretory pathway^21,22^. Thus, a longer TMD is required to reach the thicker lipid bilayer of the plasma membrane, while a rather short TMD directs proteins to the Golgi or towards the endosomal and vacuolar pathway, supporting a lipid-based sorting mechanism along the secretory pathway^18,20,23^. Even within the ER membranes, lateral heterogeneity of the lipid bilayer compartmentalizes this membrane system into different regions, including raft-like microdomains of increased lipid bilayer thickness, leading to a segregation of membrane proteins according to TMD length^19,24,25^. In yeast and mammalian cells, these ER membrane microdomains contribute to protein sorting and targeting, serve as platforms for signaling and support proper protein folding and multimer formation^1,6,10,26–31^. Interestingly, also the contact sites between the ER and mitochondria at so-called mitochondria-associated ER membranes (MAMs) represent raft-like microdomains rich in sphingolipids and sterols^32,33^, suggesting that microdomain formation contributes to interorganellar connectivity. Genomically-encoded fluorescent reporters with differing TMD length facilitated the visualization of distinct microdomains within the yeast ER membranes, marking the cortical ER membrane region at the neck of the nascent bud as raft-like microdomain with increased lipid bilayer thickness^25^. This sphingolipid-rich microdomain seems to restrict the passage of proteins with short TMD, likely contributing to the asymmetric inheritance of ER material between mother and daughter cell^25,34^. Still, subdomain features such as size, formation, lifetime, physiological regulation and dynamic remodeling depending on the cellular status remain mostly elusive.

Here, we map how microdomains in the ER membranes are rearranged in response to changing metabolic demands and cellular aging. Using recently established biosensors with differing TMD length to visualize ER lipid bilayer thickness^25^, we show that cellular age drives the formation of numerous microdomains of increased thickness particularly in the cortical ER. Moreover, we find that selectively sensors with short TMD overaccumulate at the ER membrane patches that form the nucleus-vacuole junction (NVJ), the contact site between the perinuclear ER and the vacuole that largely expands in size as cells age. In contrast, proteins with long TMD were excluded from the NVJ, suggesting that the associated ER membrane represents a subdomain with decreased lipid bilayer thickness. Partitioning into specific ER microdomains prominently affected protein turnover rates and determined whether the integral ER protein was preferentially removed via macroautophagy, microautophagy or ER-associated degradation (ERAD), all contributing to the turnover of the ER and the nucleus^35–38^. Collectively, our results show that cellular age causes a dynamic remodeling of microdomains in the ER that results in TMD-dependent partitioning to membrane contact sites and modulates protein turnover rates.

## Results

### Age-dependent remodeling of the ER membrane

**Figure 1:**
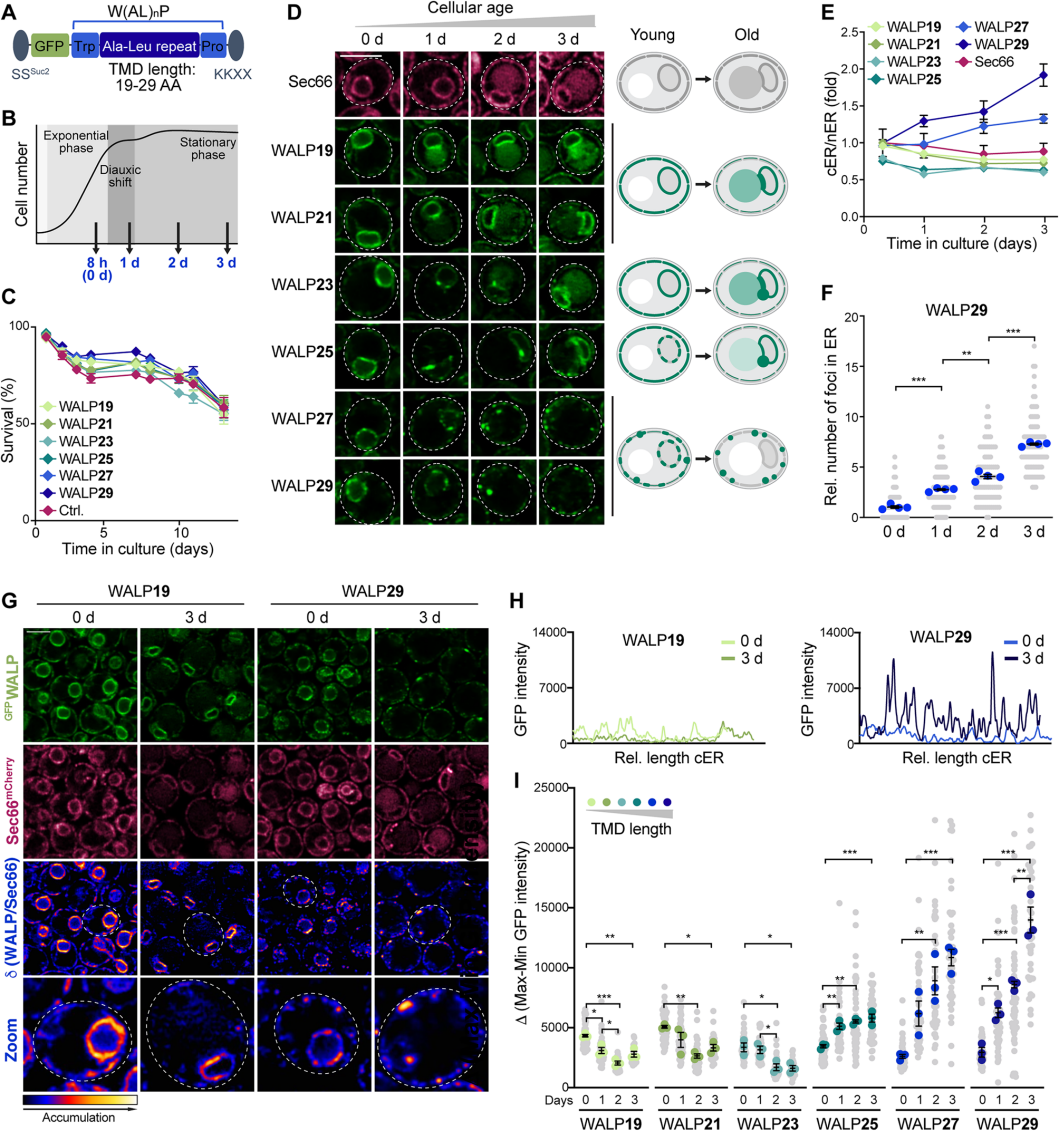
Age-dependent remodeling of the ER membrane. (**A**) Schematic of the ER membrane thickness reporters (^GFP^WALPs) based on a transmembrane domain (TMD) of varying length (19–29 AA), achieved by different numbers of Ala-Leu dipeptides. The SS^Suc2^ signal sequence facilitates ER membrane targeting and the KKXX signal retains the reporters within the ER. (**B**) Schematic illustrating the time points analyzed. Cells were assessed during exponential growth (0 day/8 h), at the end of the diauxic shift (1 day) and in early/late stationary phase (2 day and 3 day). (**C**) Flow cytometric quantification of cell death during chronological aging via propidium iodide staining of wild type (WT) and cells endogenously expressing ^GFP^WALPs. Mean ± s.e.m.; n = 4. (**D**) Micrographs of cells expressing Sec66^mCherry^ and indicated ^GFP^WALPs. Scale bar: 3 μm. Schematics of age-dependent redistribution of the WALP sensors and vacuolar GFP accumulation. (**E**) Ratio of the mean GFP intensities of the cortical (cER) and nuclear ER (nER) of cells expressing ^GFP^WALPs, quantified from confocal micrographs and depicted as fold of the WALP21 cER/nER ratio in young cells (0 d). Mean ± s.e.m.; n = 5, with > 50 cells per n. (**F**) WALP29 foci frequency, showing individual cells from 4 independent experiments (gray dots), the average for each experiment (blue dots; 17–40 cells per n), and the grand mean ± s.e.m. (lines) of the individual experiments (n = 4). (**G**) Micrographs of young and old cells expressing Sec66^mCherry^ and WALP19 or WALP29. The ratio of ^GFP^WALP to Sec66^mCherry^ visualizes the ER membrane regions with specific ^GFP^WALP accumulation. Scale bar: 3 μm. (**H**) GFP fluorescence intensity profiles of the cER of a representative cell expressing WALP19 or WALP29 at day 0 and day 3. (**I**) GFP intensity profiles as shown in (**H**) were used to calculate the average difference between the minimal and maximal GFP intensity of the cER per cell. Individual cells from 3 independent experiments (gray dots), the average for each experiment (colored dots; 10–30 cells per n), and the grand mean ± s.e.m. (lines) of the individual experiments (n = 3) are shown. *p ≤ 0.05, **p ≤ 0.01, ***p ≤ 0.001.

To assess how ER bilayer thickness and organization is affected by cellular age, we equipped yeast cells with a collection of previously established biosensors for membrane thickness (WALPs)^25^. These genomically-encoded reporters are based on a TMD of varying length (28.5 Å to 43.5 Å), achieved by a variable number of alanine-leucine dipeptide (AL)_n_ repeats, flanked by the bulky hydrophobic amino acid tryptophan (W) and a proline (P) as helix breaker. The TMD is fused to a GFP moiety to enable visualization, targeted to the ER membrane via an N-terminal signal sequence (SS^Suc2^) and retained within the ER by a classical retention signal (KKXX) (Fig. 1A). Importantly, these biosensors do not induce microdomain formation and clustering themselves^25^. They are constitutively expressed, insert efficiently into the ER membrane, do not aggregate and are highly stable^25^, thus allowing in vivo monitoring of ER bilayer organization throughout aging. The distribution of WALPs was analyzed in young, exponentially dividing cells (collected 8 h after inoculation and referred to as day 0), in cells at the end of the diauxic shift as well as in cells during early chronological aging (Fig. 1B). Survival was not affected by the endogenous expression of the reporters (Fig. 1C). Sec66, a translocon subunit with a single TMD of 21 AA, thus reflecting the average length of TMDs within the yeast ER membrane, served as reference protein. In young cells, WALP19 and WALP21 localized evenly throughout the perinuclear ER (nER) and the cortical ER (cER) patches, reminiscent of the distribution of Sec66^mCherry^ (Fig. 1D). With increasing cellular age, these WALPs remained evenly distributed but localized mainly to the nER, with a slight enrichment at the interface between the nER and the vacuole, representing the NVJ. Interestingly, slightly longer TMDs (WALP23 and WALP25) accumulated at distinct foci at the rim of the NVJs. In line with previous findings demonstrating that reporters with very long TMD specifically decorate so-called ‘regions of increased thickness’, indicative of raft-like microdomains^19,25^, we observed a punctate distribution for WALPs with long TMDs (WALP27 and WALP29) in exponentially dividing cells (Fig. 1D). Metabolic reprogramming during the switch from fermentation to respiration upon glucose depletion resulted in the accumulation of these WALPs in a few distinct foci at the nER. With progressing cellular age, those foci disappeared and WALP27 and WALP29 were mostly excluded from the nER and instead accumulated in numerous microdomains along the cER (Fig. 1D). To quantify this age-dependent change of ER membrane organization, we determined the ratio of cER to nER GFP intensities per cell for each individual WALP. While WALPs with short TMD became more abundant in the nER over time, WALP27 and in particular WALP29 progressively accumulated in the cER (Fig. 1E). Automated quantification of GFP foci per cell, visible in one focal plane, demonstrated an overall increase of the frequency of WALP29-decorated ER membrane microdomains with cellular age (Fig. 1F), though the number of these microdomains varied substantially between cells within a heterogenous clonal population. Imaging of cells simultaneously expressing ^GFP^WALP and Sec66^mCherry^ followed by calculation of the ratio of GFP to mCherry fluorescence intensities for all ER pixels confirmed that WALP19 and Sec66 signal intensities correlated well (Fig. 1G; Supplementary Fig. S1). In contrast, WALP29-enriched structures did not correspond to membrane regions with increased Sec66^mCherry^ signal, supporting that these foci indeed represent microdomains. In young cells, the WALP29-enriched microdomains of increased bilayer thickness were mainly restricted to the nER, while aged cells formed these microdomains specifically in the cER (Fig. 1G). In support of this, GFP intensity profiles of the cER revealed a prominent increase in the number and intensity of ^GFP^WALP29 foci in aged cells, while ^GFP^WALP19 remained evenly distributed (Fig. 1H). Using these intensity profiles to calculate the mean difference between minimal and maximal GFP fluorescence intensity along the cER per cell supported that shorter WALPs with TMD lengths of 19–23 AA localized evenly along the cER, while long WALPs accumulated in foci that gradually increased in intensity over time (Fig. 1I). In young, dividing cells, some of the WALP29-positive foci have been shown to correspond to ER-Golgi contact sites^25^. Thus, we tested for co-localization of WALP29 with two established markers for these contact sites: Nvj2, which mainly resides at the NVJs but redistributes to and increases ER-Golgi contact sites upon stress^39^ and Osh1, which targets both the NVJs and the ER-Golgi contact sites to support sterol transfer^40^. Microscopic analysis revealed that only a small subpopulation of the numerous WALP29-decorated foci formed during aging were juxtaposed to Nvj2^mCherry^- or Osh1^mCherry^-positive foci, while the majority corresponded to microdomains unrelated to ER-Golgi contact sites (Supplementary Fig. S2). Consistently, compromising ER-Golgi contact formation by genetic ablation of Nvj2 and Osh1 individually or simultaneously did not impact the distribution or frequency of WALP29-positive microdomains (Supplementary Fig. S3), indicating that the increased number of WALP29 foci during cellular aging is not caused by a re-organization of ER-Golgi contact sites. Collectively, this suggests a remodeling of ER lipid bilayer organization during cellular aging that drives a progressive exclusion of proteins with long TMDs from the nER and a formation of microdomains with increased bilayer thickness specifically within the cER.

### TMD length as determinant of NVJ localization

**Figure 2:**
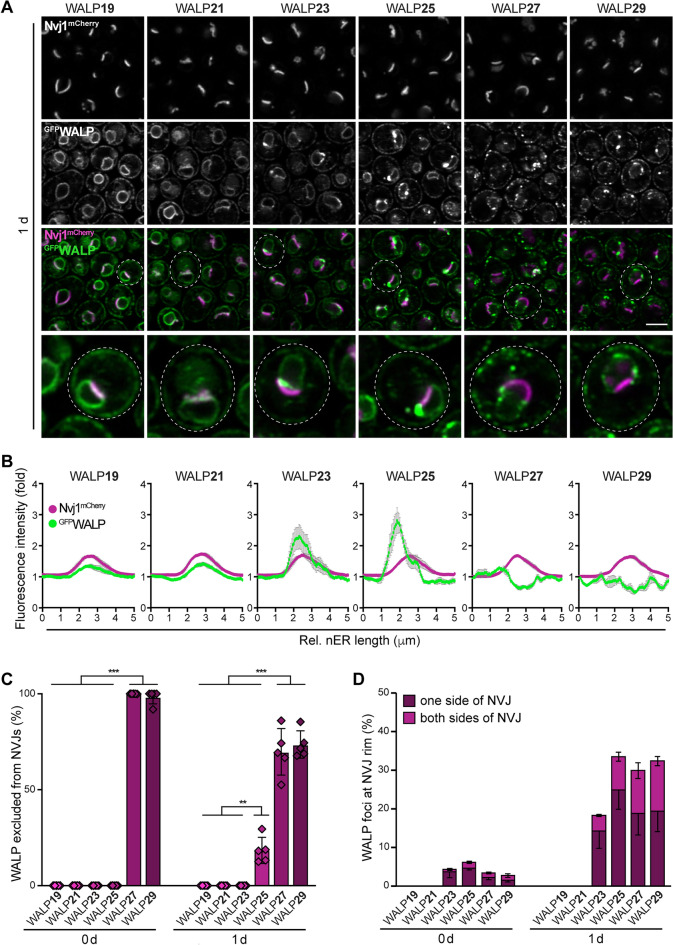
TMD length as determinant of NVJ localization. (**A**) Micrographs of cells endogenously expressing Nvj1^mCherry^ and one of the six ^GFP^WALPs at day 1 of chronological aging. Scale bar: 3 μm. (**B**) Relative GFP and mCherry fluorescence intensity profiles of the nuclear ER (nER) of cells expressing ^GFP^WALPs and Nvj1^mCherry^ from micrographs as shown in (**A**). The profiles were obtained by encircling the nER using the free-hand tool in Fiji, starting at the opposite site of the nucleus vacuole junctions (NVJs). Fluorescence intensity fold values are shown. Four individual experiments were performed, and for each experiment, profiles of 20–64 cells have been averaged. Data is depicted as mean ± s.e.m. of these individual experiments (n = 4). (**C**) Quantification of cells in which ^GFP^WALP is excluded from the NVJs at indicated time points. Data represent mean ± s.e.m.; n = 5, and at least 100 cells per n were analyzed. (**D**) Percentage of cells with ^GFP^WALP foci at the rim of the NVJ. Cells have been grouped according to ^GFP^WALP foci at only one or both sides of the NVJ. Data represent mean ± s.e.m.; n = 5, and at least 100 cells per n were analyzed. **p ≤ 0.01, ***p ≤ 0.001.

Following the diauxic shift, biosensors with shorter TMDs seemed to overaccumulate at the ER membrane regions of the NVJs, the contact sites between the vacuole and the nER. This organellar contact is established by interactions of the integral nER protein Nvj1 with the armadillo repeat-containing protein Vac8, anchored to the vacuolar membrane via palmitoylation^41^. This tethering pair provides a multi-functional platform for the recruitment of additional contact site components, most of them involved in lipid metabolism^42^. For instance, the sterol transporter Lam6 is targeted to the NVJs by association with Vac8^43^, and the recruitment of Snd3 and Osh1 to the contact sites depends on interactions with the cytoplasmic domain of Nvj1^44–46^. The enoyl-CoA reductase Tsc13 and the Hmg-CoA reductase Hmg1, two polytopic ER membrane proteins, seem to associate with Nvj1 via their transmembrane regions^47,48^. Interestingly, the sequestration of these proteins to NVJs is impacted by sterol sensing (for Hmg1)^47^ and fatty acid biosynthesis (for Tsc13)^48^, pointing to further regulatory cues that influence partitioning into NVJs. We equipped ^GFP^WALP-expressing cells with Nvj1^mCherry^ to visualize the NVJs and analyzed these cells after the diauxic shift, where NVJs prominently expand mainly due to a transcriptional upregulation of Nvj1^46,49^. WALPs with a TMD length of up to 25 AA colocalized with Nvj1^mCherry^ and decorated the NVJs, whereas a longer TMD resulted in an exclusion from the ER membrane region forming the NVJs (Fig. 2A). Fluorescence intensity profiles of the nER demonstrated that in particular WALP19, WALP21 and WALP23 overaccumulated at the NVJs, while WALP27 and WALP29 were absent (Fig. 2B). Likewise, the quantification of cells showing colocalization of ^GFP^WALP and Nvj1^mCherry^ revealed that WALP19-25 decorated the NVJs in almost all cells, while WALP27-29 were excluded from these contact sites (Fig. 2C). These biosensors only differ in the length of the TMD and are devoid of any protein domains that might facilitate specific targeting to NVJs via protein–protein or protein-lipid interaction, suggesting that partitioning of these biosensors into NVJs is determined simply by TMD length (Fig. 2B). With increasing TMD length, the reporters accumulated at distinct foci that either still co-localized with Nvj1^mCherry^ at the rim of the NVJs (WALP23 and in particular WALP25) or that were excluded from NVJs but formed juxtaposed (WALP27 and WALP29) (Fig. 2A,D). The rim of the NVJ has been suggested to contribute to lipid droplet biogenesis and organization specifically upon nutrient limitation and the subsequent diauxic shift^49,50^. As the WALP foci, reflecting the formation of specific membrane domains, appeared frequently at the elongated NVJs in cells after the diauxic shift, but were rare in exponentially growing cells, which typically display small, punctate NVJs (Fig. 2D), we assessed lipid droplet localization. Co-staining with the neutral lipid dye monodansylpentane revealed that some but not all WALP25 foci forming at the edges of the NVJs in nutrient-exhausted cells were juxtaposed to a lipid droplet (Supplementary Fig. S4). Still, the formation of these WALP25-decorated microdomains did not depend on NVJ-localized lipid droplet organization and biogenesis, as genetic ablation of Mdm1, which has been shown to be critical for lipid droplet clustering at the NVJs^49,51^, did not impact WALP25 foci formation at these sites (Supplementary Fig. S4). In sum, our data suggest that the length of the TMD can impact the localization to membrane contact sites. A short TMD supported the accumulation of the biosensors at the NVJs, though it remains to be explored how this contributes to the distribution of physiological contact site residents that are targeted to these sites depending on protein–protein interactions, various regulatory and functional domains and complex metabolic cues. Long TMDs resulted in an exclusion from the NVJs, suggesting that the ER membrane subdomains establishing this organelle contact likely represent specific regions with decreased lipid bilayer thickness. Instead, the sensors with long TMD accumulated in foci at the rim of the NVJs, potential raft-like microdomains that form specifically after the diauxic shift.

### Partitioning into raft-like microdomains increases protein stability and prevents autophagic removal

**Figure 3:**
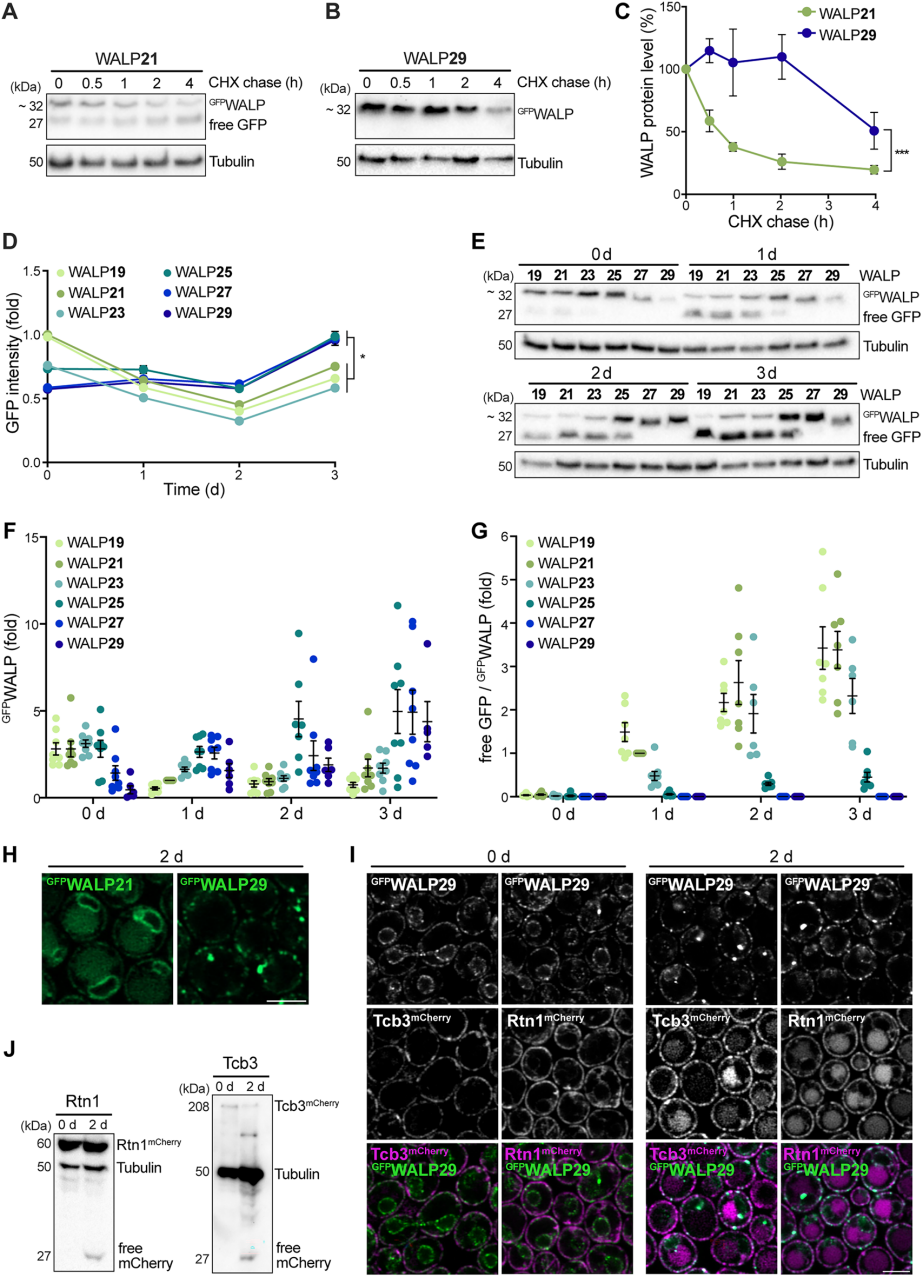
Partitioning into raft-like microdomains increases protein stability and prevents autophagic removal. (**A**–**C**) Immunoblot analysis of total protein extracts from WT cells expressing ^GFP^WALP21 or ^GFP^WALP29 collected at indicated time points. After 8 h of growth, cells were treated with cycloheximide (CHX) to stop translation and the turnover of ^GFP^WALP was determined. Representative blots (**A**, **B**) and corresponding densitometric quantification of the ^GFP^WALP protein levels (**C**) are shown. Blots were probed with antibodies directed against GFP and tubulin as loading control. The ^GFP^WALP protein levels are shown as percentage of protein level before CHX treatment (0 h); Data represent mean ± s.e.m.; n = 5. (**D**) Flow cytometric quantification of total cellular GFP fluorescence intensity of WT cells expressing one of the six ^GFP^WALPs at indicated time points. Dead cells were excluded from the analysis via counterstaining with propidium iodide. GFP intensity is shown as fold of WALP21 at day 0; Data represent mean ± s.e.m.; n = 6. (**E**–**G**) Immunoblot analysis of total protein extracts from WT cells expressing one of the six ^GFP^WALPs collected at indicated time points. Blots were probed with antibodies directed against GFP and tubulin as loading control. A representative blot (**E**) and corresponding densitometric quantification of ^GFP^WALP protein levels normalized to tubulin (**F**) as well as of the ratio of free GFP to ^GFP^WALP, indicative of autophagic turnover (**G**), are shown. Values have been normalized to the respective values of ^GFP^WALP21 at day 1. Dot plots with mean ± s.e.m.; n = 8. (**H**) Micrographs of cells expressing ^GFP^WALP21 or ^GFP^WALP29 at day 2 of chronological aging. Scale bar: 3 μm. (**I**) Micrographs of cells expressing ^GFP^WALP29 and either Tcb3^mCherry^ or Rtn1^mCherry^ at indicated days during aging. Scale bar: 3 μm. (**J**) Immunoblot analysis of total protein extracts from cells described in (I). Blots were probed with antibodies directed against mCherry and tubulin as loading control. *p ≤ 0.05, ***p ≤ 0.001.

The turnover rates of proteins differ substantially and are impacted by numerous factors, such as protein localization, abundance, size, assembly into complexes, and the general cellular state^52–54^. Global determination of protein turnover rates in exponentially dividing yeast cells has revealed an average half-life of around 2.3 h for ER proteins^54^. To test whether the TMD length of ER-resident proteins affects stability and turnover rates, we assessed WALP21 and WALP29 protein levels following inhibition of de novo protein biosynthesis by cycloheximide. WALP21 was rapidly degraded, with a half-life of less than 1 h, while WALP29 was rather stable, displaying a half-life of around 4 h (Fig. 3A–C). Flow cytometric determination of cellular GFP intensities during aging revealed an accumulation of ^GFP^WALPs with TMDs longer than 25 AA over time (Fig. 3D). In line, immunoblotting demonstrated that the levels of WALPs with longer TMDs gradually increased with cellular age, while the levels of WALPs with shorter TMDs dropped over time (Fig. 3E,F). Thus, partitioning into microdomains of increased bilayer thickness protects ER membrane proteins from proteolytic removal. Integral ER proteins can be removed via ER-associated degradation (ERAD), facilitating their retrotranslocation to the cytosol for subsequent degradation by the proteasome, or via autophagy, resulting in protein breakdown upon delivery to the vacuole^35–38^. Autophagic removal of GFP-tagged proteins results in the accumulation of free GFP, as the GFP moiety is rather resistant to vacuolar hydrolysis, allowing the quantification of autophagic protein removal. We observed a time-dependent increase of free GFP specifically in cells expressing WALPs with shorter TMDs (Fig. 3E,G). In contrast, free GFP was completely absent in cells expressing WALP27 and WALP29 throughout the aging. Consistently, microscopic analysis demonstrated that WALP21 but not WALP29 was delivered to the vacuole for subsequent breakdown (Fig. 3H), suggesting that a long TMD prevents WALP turnover via autophagy. This was not due to the age-dependent redistribution of WALP29 foci from the nER to the cER, as other proteins that predominantly reside within the cER were accessible to autophagic turnover (Fig. 3I,J). The ER-shaping reticulon protein Rtn1 and the tricalbin Tcb3, an ER-plasma membrane tethering protein, have been shown to almost exclusively localize to the cER and to insert into the membrane via hairpin-like transmembrane domains^55–58^. Microscopic analysis of endogenously expressed Rtn1^mCherry^ and Tcb3^mCherry^ revealed that these cER proteins were not specifically enriched at WALP29-decorated microdomains (Fig. 3I), though a subpopulation of Tcb3-marked ER-plasma membrane contacts correlated with WALP29 foci, indicating a thicker bilayer at these sites. Still, Tcb3 in addition localized to WALP29-negative membrane regions. Both Tcb3^mCherry^ and Rtn1^mCherry^ were subjected to autophagic breakdown, indicated by the age-dependent appearance of vacuolar mCherry signal (Fig. 3I). In line, immunoblotting demonstrated mCherry liberation from Rtn1^mCherry^ and Tcb3^mCherry^ in stationary phase, suggesting autophagic delivery to and turnover in the vacuole (Fig. 3J). Collectively, this suggests that not the age-induced targeting of WALP29 to the cER but rather the prominent partitioning into raft-like microdomains prevents removal via autophagy.

### Reporters with short TMD are degraded via piecemeal microautophagy of the nucleus

**Figure 4:**
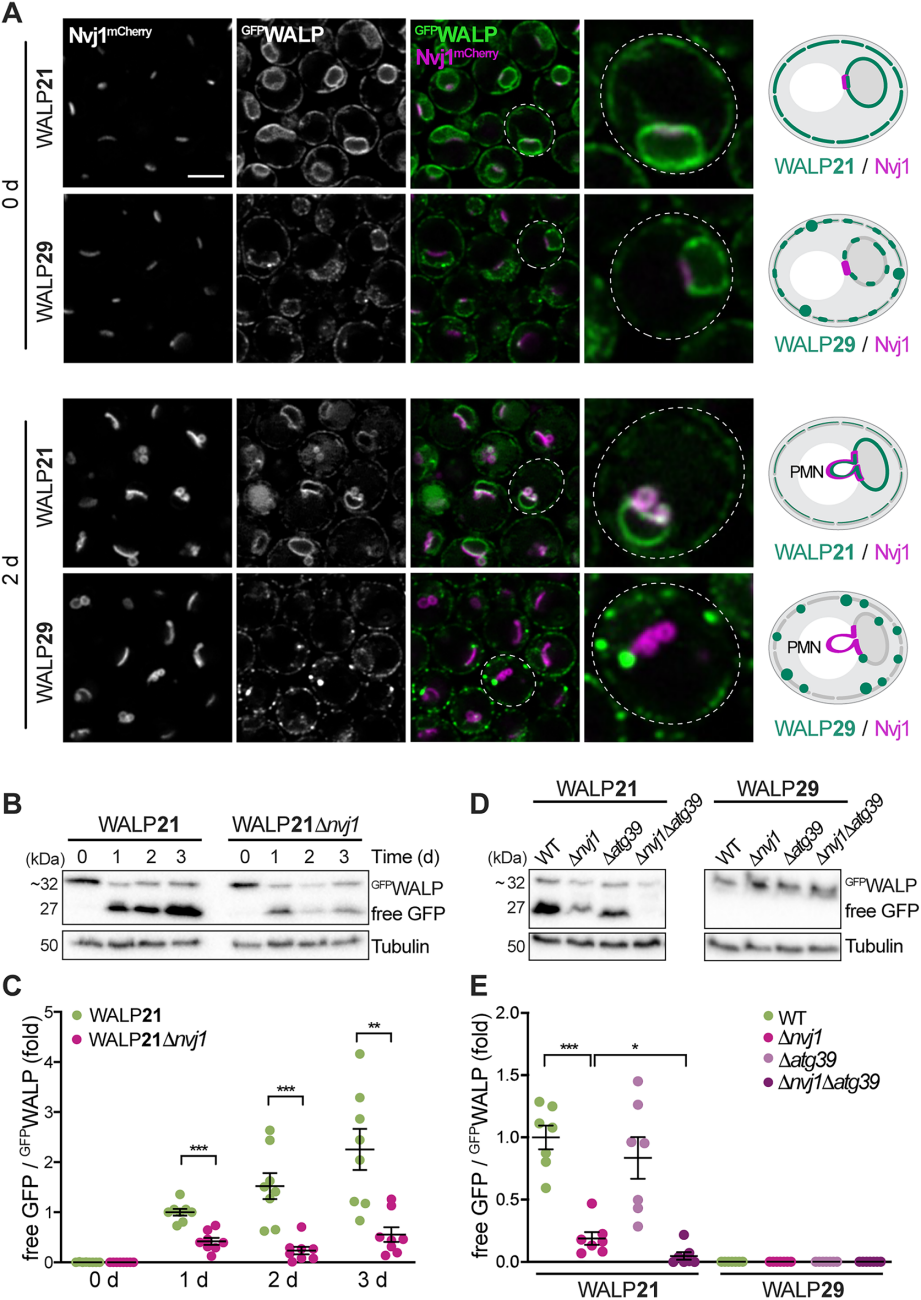
Reporters with short TMD are removed via piecemeal microautophagy of the nucleus. (**A**) Micrographs of exponentially growing and stationary cells endogenously expressing Nvj1^mCherry^ and ^GFP^WALP21 or ^GFP^WALP29. Scale bar: 3 μm. Schematics of TMD length-dependent exclusion of WALP29 from the nucleus vacuole junction (NVJ) and piecemeal microautophagy of the nucleus (PMN). (**B**, **C**) Immunoblot analysis of total protein extracts from wild type (WT) and Δ*nvj1* cells expressing ^GFP^WALP21 collected at indicated time points during chronological aging. A representative blot (**B**) and corresponding densitometric quantification of the ratio of free GFP to ^GFP^WALP21 (**C**) are depicted. Blots were decorated with antibodies directed against GFP and tubulin as loading control. The free GFP/^GFP^WALP values are shown as fold of the ratio at day 1. Dot plots with mean ± s.e.m.; n = 8. (**D**, **E**) Immunoblot analysis of total protein extracts from WT, Δ*nvj1*, Δ*atg39*, and Δ*nvj1*Δ*atg39* cells expressing ^GFP^WALP21 or ^GFP^WALP29 collected at day 2. Representative blots (**D**) as well as corresponding densitometric quantification of the ratio of free GFP to ^GFP^WALP (**E**) are depicted. Blots were probed with antibodies directed against GFP and tubulin as loading control. The free GFP/^GFP^WALP values are shown as fold of the ratio of ^GFP^WALP21 in WT. Dot plots with mean ± s.e.m.; n = 7. *p ≤ 0.05, **p ≤ 0.01, and ***p ≤ 0.001.

Bulk degradation of ER membrane proteins occurs via micro- and macroautophagic processes. Macroautophagy of parts of the ER and the continuous nuclear membranes, termed ER-phagy and nucleophagy, is mediated by the selective autophagy receptors Atg39 and Atg40, which interact with Atg8 to facilitate autophagosome formation and cargo engulfment^36,37,59^. Microautophagic degradation of portions of the nuclear and nER membranes, so-called piecemeal microautophagy of the nucleus (PMN), occurs only at the NVJs and refers to the pinching-off and release of PMN-vesicles into the vacuolar lumen^38,60^. As WALPs with short TMDs overaccumulated at the NVJs in post-diauxic shift cells, we tested whether this primes for degradation via PMN using Nvj1^mCherry^ as marker for NVJs and associated micronucleophagy. Indeed, ^GFP^WALP21 (but not ^GFP^WALP29) decorated the nER blebs that emerged when cells reached stationary phase, budding into the vacuole (Fig. 4A). In line with the notion that degradation via PMN occurs specifically upon nutrient exhaustion, PMN vesicles were absent in cells growing exponentially in glucose-rich media (day 0). Immunoblotting and quantification of vacuolar GFP liberation from ^GFP^WALP21 confirmed prominent autophagic degradation following the switch from fermentative to respiratory metabolism upon glucose depletion (Fig. 4B,C). Genetic ablation of Nvj1 largely reduced the levels of free GFP, demonstrating that the autophagic turnover of ^GFP^WALP21 requires NVJ formation and mainly occurs via PMN (Fig. 4B,C). The residual GFP liberation in cells lacking Nvj1, indicative of low levels of autophagic degradation via alternative mechanisms, was completely blocked by additional deletion of *ATG39* to inhibit nucleophagy (Fig. 4D,E). Thus, a short TMD results in enrichment of the reporter at the NVJs and subsequent autophagic removal mainly via PMN and, to a minor extent, also Atg39-mediated macronucleophagy. Although simultaneous inactivation of macro- and micronucleophagy completely prevented autophagic turnover of ^GFP^WALP21, this did not result in a prominent accumulation of the reporter, suggesting removal by alternative means when nucleophagy is blocked. Vacuolar GFP liberation was absent for ^GFP^WALP29 (Fig. 4D,E), demonstrating that a long TMD, which drives partitioning into raft-like microdomains, prevents removal from the ER via nucleophagic processes.

### TMD-independent degradation of ER membrane proteins via ERAD

**Figure 5:**
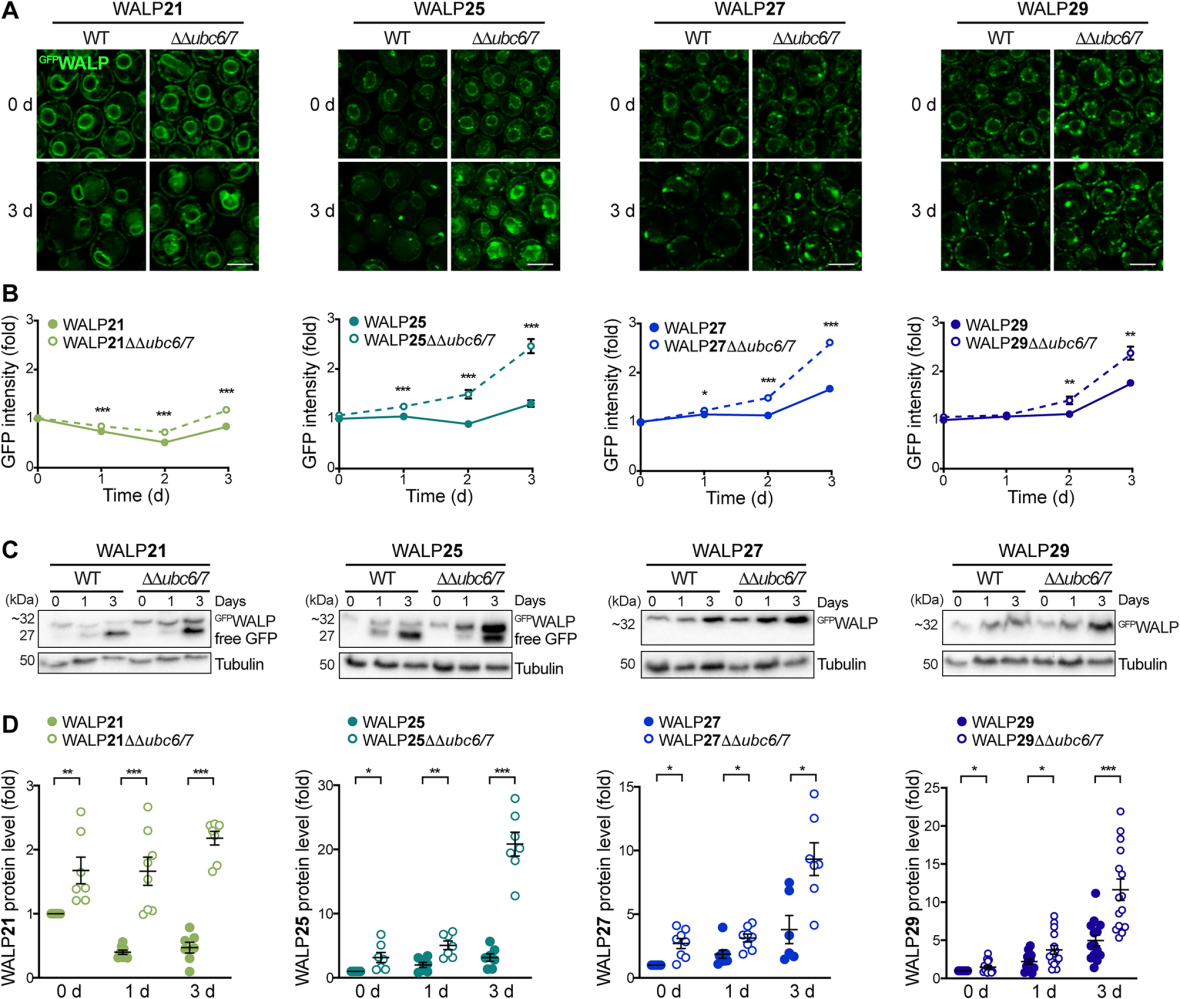
TMD-independent degradation of ER proteins via ERAD. (**A**) Micrographs of young and old wild type (WT) cells and cells lacking the two ubiquitin-conjugating enzymes Ubc6 and Ubc7 (ΔΔ*ubc6*/7), endogenously expressing ^GFP^WALP21, ^GFP^WALP25, ^GFP^WALP27, or ^GFP^WALP29. Scale bar: 3 μm. (**B**) Flow cytometric quantification of GFP fluorescence intensity of WT and ΔΔ*ubc6*/7 cells expressing indicated ^GFP^WALPs. Dead cells were excluded from the analysis via counterstaining with propidium iodide. Data represent mean ± s.e.m.; n = 8. (**C**, **D**) Immunoblot analysis of total protein extracts from WT and ΔΔ*ubc6/7* cells expressing ^GFP^WALP21, ^GFP^WALP25, ^GFP^WALP27, or ^GFP^WALP29 collected at indicated time points. Representative blots (**C**) as well as corresponding densitometric quantification of the ^GFP^WALP protein levels (**D**) are shown. Blots were probed with antibodies directed against GFP and tubulin as loading control. ^GFP^WALP protein levels were normalized to tubulin and are shown as fold of the respective 0 day time point. Dot plots with mean ± s.e.m.; *p ≤ 0.05, **p ≤ 0.01, ***p ≤ 0.001.

To assess the contribution of ER-associated degradation (ERAD) to the removal of WALPs, we genetically inactivated ERAD via deletion of the genes coding for the ubiquitin-conjugating enzymes Ubc6 and Ubc7^35,61^. Contrary to nucleophagic degradation of GFP-tagged proteins, no free GFP will be generated upon retrotranslocation of clients from the ER membrane via ERAD and subsequent proteasomal degradation. Confocal microscopy revealed that all WALPs were stabilized to some extend upon loss of ERAD (Fig. 5A). Likewise, flow cytometric evaluation of total cellular GFP intensity as well as determination of protein level using immunoblotting showed an overall accumulation of ^GFP^WALPs over time in cells with inactivated ERAD (Fig. 5B–D). Hence, our results suggest that all biosensors can be degraded via ERAD independent of their TMD length, while the partitioning of sensors with long TMD into microdomains of increased bilayer thickness prevents their turnover via autophagy.

## Discussion

A cell adapts to changing metabolic needs by a prominent reorganization of numerous cellular structures, networks and processes. Upon nutrient limitation, yeast cells exit cell cycle, enter a non-proliferative, stationary state characterized by increased stress tolerance and undergo chronological aging. The redirection of resources from the biosynthesis of macromolecules to support rapid cell growth towards maintenance, storage and the induction of stress response pathways requires remodeling of organelle function and connectivity.

Here, we provide first insights into the rearrangement of microdomains within organellar membranes in response to metabolic reprogramming and cellular aging. In the ER membrane of young, proliferating yeast cells, raft-like microdomains mainly exist within the nER. With the shift to a respiratory metabolism, entry into a non-dividing state and progressing age, these nER microdomains disappear, and the cells instead form numerous microdomains in the cER. This might reflect adaptation to changing anabolic demands within the ER network, which serves as hub for lipid synthesis and protein biogenesis. The formation of distinct lateral microdomains in the ER is assumed to be critical for protein sorting within the secretory pathway, where not only protein–protein interactions, depending on specific sequences and sorting machineries, but also the interaction between the TMD and the lipid bilayer contribute to protein partitioning into specific platforms and correct protein trafficking^62^. Similar to the plasma membrane, association with ER-localized raft-like microdomains, either via glycosylphosphatidylinositol (GPI)-anchor or TMD, restricts lateral diffusion of the protein^63^. Chronological aging in yeast is associated with a restructuring and thickening of the cell wall and thus an increased demand for proteins associated with its synthesis, often GPI-anchored surface proteins associated with plasma membrane lipid rafts such as Gas1^64^. Sorting of these surface proteins into sphingolipid-rich rafts has been shown to already start within the ER membrane and to be essential for efficient delivery to the plasma membrane^26^. Similarly, the plasma membrane-localized proton pumping ATPase Pma1, necessary to maintain pH homeostasis, is sorted into raft-like microdomains already within the ER^26^. Thus, the increased frequency of these domains within the ER membrane as cells age might contribute to the rearrangement of the protein composition of the plasma membrane. This increased capacity to sort and deliver different surface proteins via raft-like platforms could, for instance, support age-associated cell wall biosynthesis, tailor nutrient uptake and counteract cytosolic acidification, which is linked to premature cellular demise during chronological aging^65,66^.

Beyond their function in protein sorting, raft-like microdomains in the ER have also been implicated in the formation of membrane contact sites. Mitochondria-associated ER membranes, the MAMs, have been shown to represent raft-like microdomains^32,33^. Similarly, the membrane contact sites between the ER and the plasma membrane have been suggested to exhibit raft-like characteristics^67^. We find that a subpopulation of Tcb3-demarcated ER-plasma membrane contact sites correlates with WALP29 foci, in particular in aged cells. Whether the increased number of microdomains in the cER that we observed in aged yeast cells is a response to enhanced ER-plasma membrane tethering remains to be investigated. As these contacts are linked to lipid metabolism^68,69^, ER stress surveillance^70^ and general ER function and morphology^71^, the rearrangement of the microdomains within the cER might support cellular adaptation to changing anabolic and catabolic needs during aging. Interestingly, we find that the extended ER membrane patches in contact with the vacuole at NVJs represent distinct microdomains that do not resemble lipid rafts. While biosensors with long TMDs were excluded from the NVJs, those with short TMDs freely passed and even overaccumulated at the extended NVJ patches as cells aged, suggesting that the associated ER membrane represents a subdomain with decreased bilayer thickness.

Importantly, we find that the length of the TMD and thus partitioning into distinct microdomains impacts how fast and by which degradative pathway the artificial ER membrane protein will be removed. Biosensors with short TMD were turned over rather quickly, and protein levels dropped as cells aged, mainly due to the combined action of selective micro- and macroautophagy. While a small portion of theses WALPs was removed by Atg39-mediated nucleophagy, their overaccumulation at the large NVJ patches in aging cells prominently targeted them for microautophagic degradation via PMN. Our results indicate that the ER membrane at this contact site likely represents a thinner and thus more flexible membrane subdomain, which might prime this region for the frequent deformation and budding-off of PMN vesicles into the vacuole occurring in stationary cells. In contrast, a long TMD and the associated clustering in raft-like microdomains increased the biosensor half-life about fourfold and resulted in gradually increasing protein levels over time. Notably, these microdomain-associated biosensors were excluded from age-induced autophagic degradation of the ER membrane system. The removal of ER and nuclear material via selective macroautophagy requires cargo sequestration by specific transmembrane receptors and subsequent membrane protrusion towards the cytosol before packing into autophagosomes^72,73^. The thicker and stiffer raft-like microdomains might impede receptor-mediated deformation of the ER membrane and thus preclude cargo turnover via selective autophagy.

Raft-like microdomains most prominently form in the plasma membrane but likely exist in most intracellular membranes as well, including not only the ER but also the Golgi, lysosomes/vacuoles and also mitochondria^1^. Proteomic profiling of detergent-resistant membranes, corresponding to raft-like microdomains, in *Candida albicans* revealed that numerous proteins that partitioned into these domains were linked to mitochondria, Golgi, and the ER^74^. Though the existence of specific microdomains that enable further subcompartmentalization seems to be characteristic for most biological membranes^1^, the physiological relevance, regulation, molecular composition, size and dynamics of these clusters remain largely unexplored. Our results suggest a prominent rearrangement of distinct ER microdomains during metabolic adaptation and cellular aging that impacts on protein targeting to membrane contact sites and autophagic protein turnover.

## Materials and methods

### Yeast strains and genetics

All experiments were performed using *S. cerevisiae* BY4741 (*MATa*, *his3*Δ1, *leu2*Δ0, *met15*Δ0, *ura3*Δ0). Yeast transformation was carried out following established protocols^75^, and deletion or endogenous tagging was performed via homologous recombination as previously described^76^. All yeast strains, plasmids and oligonucleotides used in this study are listed in Supplementary Tables S1 and S2, respectively. For yeast transformations, complex medium (YPD) containing 20 g/L peptone (Gibco™ Bacto™ BD Biosciences, 16219761), 10 g/L yeast extract (Bacto™ BD Biosciences, 288620) and 4% glucose was used, and positive transformants were selected on respective selection media. At least three transformants were analyzed for all strains to rule out clonogenic variation.

### Media and culturing conditions

Strains were grown at 28 °C, shaking at 145 rpm in synthetic complete medium (SC) containing 0.17% yeast nitrogen base (BD Difco™, 233520), 0.5% (NH_4_)_2_SO_4_ (Carl Roth, 3746.3) and 30 mg/L of all amino acids (except 80 mg/l histidine and 200 mg/L leucine), 30 mg/L adenine and 320 mg/L uracil, with 2% glucose (SCD). Overnight cultures incubated for 16–18 h in SCD were used to inoculate cultures to OD_600_ 0.1 in 15 mL or 20 mL fresh SCD in 100 mL baffled Erlenmeyer flasks. Unless stated otherwise, samples were collected at exponential phase (8 h = 0 day), the end of the diauxic shift (24 h = 1 day), early stationary phase (48 h = 2 day) as well as late stationary phase (72 h = 3 day).

### Flow cytometric analyses

The relative GFP intensity of ^GFP^WALP-expressing cells was assessed via flow cytometry using a Guava® easyCyte 5HT with a 488 nm laser and the following emission filters: 488/16 (SSC), 525/30 (green), 605/50 (red). To exclude dead cells, cells were stained with propidium iodide (Sigma-Aldrich, 81845), which accumulates in cells that lost their membrane integrity, as previously described^77^. Briefly, approximately 1 × 10^6^ cells were harvested in a 96-well plate and incubated in phosphate buffered saline (PBS, 25 mM potassium phosphate; 0.9% NaCl; adjusted to pH 7.2) containing 500 ng/mL of propidium iodide for 10 min in the dark. Cells were pelleted, washed once in PBS and the GFP intensity per cell was recorded. Per sample, 5000 events were evaluated. Data were analyzed with InCyte™ software (3.1), and propidium iodide-positive cells were excluded.

### Immunoblot analysis

3 OD_600_ of cells were harvested at indicated time points, resuspended in 150 μL lysis buffer (1.85 M NaOH; 7.5% 2-mercaptoethanol) and incubated on ice for 10 min, with a brief vortexing after 5 min. Then, 150 μL 55% TCA was added, samples were vortexed and incubated on ice for another 10 min, with a brief vortexing after 5 min. The samples were centrifuged (10 min, 10,000×*g* at 4 °C), the supernatant was removed and the pellet was resuspended in 75 μL 1 × Laemmli buffer (0.3 M Tris Base, 12% SDS, 70% glycerol, 4% 2-mercaptoethanol, 9 mM bromophenol blue) and boiled at 95 °C for 5 min. 12 μL of protein extract were loaded on 12.5% SDS-acrylamide gels using Tris–glycine running buffer (25 mM Tris Base; 200 mM glycine; 0.05% SDS). Proteins were separated by electrophoresis at 200 mA and blotted onto PVDF membranes (ROTH, T830.1) using wet electro-transfer protocols (220 V for 60 min). After blotting, proteins were fixed to the membrane using acetone fixation^78^. To this end, membranes were immediately incubated in acetone at 4 °C for 30 min and then dried at 50 °C for another 30 min. Membranes were reactivated in ethanol for some seconds before blocking for 1 h in 5% milk powder solubilized in TBS (500 mM Tris; 1.5 M NaCl; pH 7.4). Membranes were incubated with primary antibodies against the GFP-epitope (dilution 1:2500, mouse, Sigma-Aldrich, 1181446001), the mCherry-epitope (dilution 1:1000, rabbit, Abcam, AB167453) and tubulin (dilution 1:10,000, rabbit, Abcam, 184970) and respective peroxidase-conjugated secondary antibodies against mouse (dilution 1:10,000, rabbit, Sigma-Aldrich, A9044) or rabbit (dilution 1:10,000, goat, Sigma-Aldrich, A0545). For the detection on a ChemiDocXRS + Imaging System (BIO-RAD, 1708265), Clarity Western ECL Substrate (BIO-RAD, 1705060) was used. Subsequent densitometric quantification was performed using the ImageLab 5.2.1 Software (BIO-RAD, 1709690). Full-length immunoblots including molecular weight markers are shown in Supplementary Fig. S5.

### Cycloheximide chase assay

To determine the stability of WALP21 and WALP29, yeast cells expressing either ^GFP^WALP21 or ^GFP^WALP29 were inoculated to OD_600_ 0.1, incubated for 8 h and treated with 10 mg/L cycloheximide to block protein biosynthesis. Samples were collected at indicated time points, lysed and assessed via immunoblotting as described above.

### Confocal microscopy

For confocal microscopy cells were harvested, immobilized on agar slides (3% agar in PBS), and visualized using an LSM800 Airyscan confocal microscope (ZEISS) equipped with an 63 ×/1.40 oil objective. To visualize lipid droplets, cells were resuspended in 250 μL PBS containing 100 μM monodansylpentane (MDH) (#SM1000a, AUTODOT, Abcepta), incubated for 10 min in the dark and washed with PBS prior to immobilization on agar slides. All images were analyzed and processed using the open-source software Fiji^79^. To reduce image noise, Gaussian filtering (σ = 1–2) and background subtraction (rolling ball radius = 50–150 pixel) was applied. Pictures within one experiment were taken and processed in the same way. To visualize the accumulation of a specific WALP in respect to a control ER membrane protein with average TMD length, the ratio of ^GFP^WALP to Sec66^mCherry^ per pixel was calculated and visualized using a look-up table that depicts regions of WALP overaccumulation in respect to Sec66. The quantification of WALP29 foci in the ER membrane, refering to regions of WALP29 overaccumlation in respect to Sec66 as reference protein and indicative of regions of increased bilayer thickness, was performed semi-automatically. All cells were encircled manually, followed by automated quantification of regions of increased bilayer thickness per cell, visible in one focal plane.

### Statistical analysis and data preparation

Data were analyzed and graphs were generated using GraphPad Prism 8 and 9, and figures were prepared in Adobe Illustrator. Results are shown as dot plots, superplots or line graphs with means of individual experiments and error bars representing standard error of mean (s.e.m.). Comparisons were performed using unpaired, parametric Welch t-tests (Figs. 4C,E, 5B,D), a one-way ANOVA followed by Tukey’s multiple comparison test (Figs. 1F,I, 2C), or a two-way ANOVA followed by Sidak’s multiple comparison test (Fig. 3C,D). Significances are indicated with asterisks: *p ≤ 0.05, **p ≤ 0.01, and ***p ≤ 0.001. Sample size is provided in the corresponding figure legend.

### Supplementary Information

Supplementary Information.

## Data Availability

The data generated and used in this study will be made available by the corresponding author upon reasonable request.

## References

[1] Wang H-Y, Bharti D, Levental I (2020). Membrane heterogeneity beyond the plasma membrane. Front. Cell Dev. Biol..

[2] Levental I, Levental KR, Heberle FA (2020). Lipid rafts: Controversies resolved, mysteries remain. Trends Cell Biol..

[3] Lingwood D, Simons K (2010). Lipid rafts as a membrane-organizing principle. Science.

[4] Simons K, Ikonen E (1997). Functional rafts in cell membranes. Nature.

[5] Pike LJ (2006). Rafts defined: A report on the keystone symposium on lipid rafts and cell function. J. Lipid Res..

[6] Jacobson K, Mouritsen OG, Anderson RGW (2007). Lipid rafts: At a crossroad between cell biology and physics. Nat. Cell Biol..

[7] Simons K, van Meer G (1988). Lipid sorting in epithelial cells. Biochemistry.

[8] Kinnun JJ, Bolmatov D, Lavrentovich MO, Katsaras J (2020). Lateral heterogeneity and domain formation in cellular membranes. Chem. Phys. Lipids.

[9] van Meer G, Voelker DR, Feigenson GW (2008). Membrane lipids: Where they are and how they behave. Nat. Rev. Mol. Cell Biol..

[10] Browman DT, Resek ME, Zajchowski LD, Robbins SM (2006). Erlin-1 and erlin-2 are novel members of the prohibitin family of proteins that define lipid-raft-like domains of the ER. J. Cell Sci..

[11] Ghaemmaghami S (2003). Global analysis of protein expression in yeast. Nature.

[12] Lang S (2022). Signal peptide features determining the substrate specificities of targeting and translocation components in human ER protein import. Front. Physiol..

[13] The UniProt Consortium (2017). UniProt: The universal protein knowledgebase. Nucleic Acids Res..

[14] Von Heijne G (2007). The membrane protein universe: What’s out there and why bother?. J. Internal Med..

[15] Guna A, Hegde RS (2018). Transmembrane domain recognition during membrane protein biogenesis and quality control. Curr. Biol..

[16] White SH, von Heijne G (2005). Transmembrane helices before, during, and after insertion. Curr. Opin. Struct. Biol..

[17] Dukhovny A, Yaffe Y, Shepshelovitch J, Hirschberg K (2009). The length of cargo-protein transmembrane segments drives secretory transport by facilitating cargo concentration in export domains. J. Cell Sci..

[18] Rayner JC, Pelham HR (1997). Transmembrane domain-dependent sorting of proteins to the ER and plasma membrane in yeast. EMBO J..

[19] Ronchi P, Colombo S, Francolini M, Borgese N (2008). Transmembrane domain–dependent partitioning of membrane proteins within the endoplasmic reticulum. J. Cell Biol..

[20] Singh S, Mittal A (2016). Transmembrane domain lengths serve as signatures of organismal complexity and viral transport mechanisms. Sci. Rep..

[21] Bretscher MS, Munro S (1993). Cholesterol and the golgi apparatus. Science.

[22] Schneiter R (1999). Electrospray ionization tandem mass spectrometry (Esi-Ms/Ms) analysis of the lipid molecular species composition of yeast subcellular membranes reveals acyl chain-based sorting/remodeling of distinct molecular species en route to the plasma membrane. J. Cell Biol..

[23] Sharpe HJ, Stevens TJ, Munro S (2010). A comprehensive comparison of transmembrane domains reveals organelle-specific properties. Cell.

[24] Bonnon C, Wendeler MW, Paccaud J-P, Hauri H-P (2010). Selective export of human GPI-anchored proteins from the endoplasmic reticulum. J. Cell Sci..

[25] Prasad R, Sliwa-Gonzalez A, Barral Y (2020). Mapping bilayer thickness in the ER membrane. Sci. Adv..

[26] Bagnat M, Keränen S, Shevchenko A, Shevchenko A, Simons K (2000). Lipid rafts function in biosynthetic delivery of proteins to the cell surface in yeast. Proc. Natl. Acad. Sci..

[27] Campana V (2006). Detergent-resistant membrane domains but not the proteasome are involved in the misfolding of a PrP mutant retained in the endoplasmic reticulum. J. Cell Sci..

[28] Lee MCS, Hamamoto S, Schekman R (2002). Ceramide biosynthesis is required for the formation of the oligomeric H+-ATPase Pma1p in the yeast endoplasmic reticulum. J. Biol. Chem..

[29] Muñiz M, Riezman H (2000). Intracellular transport of GPI-anchored proteins. EMBO J..

[30] Schuck S, Simons K (2004). Polarized sorting in epithelial cells: Raft clustering and the biogenesis of the apical membrane. J. Cell Sci..

[31] Simons K, Toomre D (2000). Lipid rafts and signal transduction. Nat. Rev. Mol. Cell Biol..

[32] Garofalo T (2016). Evidence for the involvement of lipid rafts localized at the ER-mitochondria associated membranes in autophagosome formation. Autophagy.

[33] Hayashi T, Fujimoto M (2010). Detergent-resistant microdomains determine the localization of sigma-1 receptors to the endoplasmic reticulum-mitochondria junction. Mol. Pharmacol..

[34] Clay L (2014). A sphingolipid-dependent diffusion barrier confines ER stress to the yeast mother cell. Elife.

[35] Carvalho P, Goder V, Rapoport TA (2006). Distinct ubiquitin-ligase complexes define convergent pathways for the degradation of ER proteins. Cell.

[36] Mochida K (2015). Receptor-mediated selective autophagy degrades the endoplasmic reticulum and the nucleus. Nature.

[37] Molinari M (2021). ER-phagy responses in yeast, plants, and mammalian cells and their crosstalk with UPR and ERAD. Dev. Cell.

[38] Roberts P (2003). Piecemeal microautophagy of nucleus in Saccharomyces cerevisiae. Mol. Biol. Cell.

[39] Liu L-K, Choudhary V, Toulmay A, Prinz WA (2017). An inducible ER-Golgi tether facilitates ceramide transport to alleviate lipotoxicity. J. Cell Biol..

[40] Shin JJH (2020). pH biosensing by PI4P regulates cargo sorting at the TGN. Dev. Cell.

[41] Pan X (2000). Nucleus-vacuole junctions in Saccharomyces cerevisiae are formed through the direct interaction of Vac8p with Nvj1p. Mol. Biol. Cell.

[42] Kohler V, Büttner S (2021). Remodelling of nucleus-vacuole junctions during metabolic and proteostatic stress. Contact.

[43] Murley A (2015). Ltc1 is an ER-localized sterol transporter and a component of ER-mitochondria and ER-vacuole contacts. J. Cell Biol..

[44] Kvam E, Goldfarb DS (2004). Nvj1p is the outer-nuclear-membrane receptor for oxysterol-binding protein homolog Osh1p in *Saccharomyces cerevisiae*. J. Cell Sci..

[45] Manik MK, Yang H, Tong J, Im YJ (2017). Structure of yeast OSBP-related protein osh1 reveals key determinants for lipid transport and protein targeting at the nucleus-vacuole junction. Structure.

[46] Tosal-Castano S (2021). Snd3 controls nucleus-vacuole junctions in response to glucose signaling. Cell Rep..

[47] Rogers S, Hariri H, Wood NE, Speer NO, Henne WM (2021). Glucose restriction drives spatial reorganization of mevalonate metabolism. Elife.

[48] Kvam E, Gable K, Dunn TM, Goldfarb DS (2005). Targeting of Tsc13p to nucleus-vacuole junctions: A role for very-long-chain fatty acids in the biogenesis of microautophagic vesicles. Mol. Biol. Cell.

[49] Hariri H (2018). Lipid droplet biogenesis is spatially coordinated at ER-vacuole contacts under nutritional stress. EMBO Rep..

[50] Henne WM, Hariri H (2018). Endoplasmic reticulum-vacuole contact sites ‘bloom’ with stress-induced lipid droplets. Contact.

[51] Hariri H (2019). Mdm1 maintains endoplasmic reticulum homeostasis by spatially regulating lipid droplet biogenesis. J. Cell Biol..

[52] Belle A, Tanay A, Bitincka L, Shamir R, O’Shea EK (2006). Quantification of protein half-lives in the budding yeast proteome. Proc. Natl. Acad. Sci. USA.

[53] Doherty MK, Hammond DE, Clague MJ, Gaskell SJ, Beynon RJ (2009). Turnover of the human proteome: Determination of protein intracellular stability by dynamic SILAC. J Proteome Res..

[54] Martin-Perez M, Villén J (2017). Determinants and regulation of protein turnover in yeast. Cell Syst..

[55] Voeltz GK, Prinz WA, Shibata Y, Rist JM, Rapoport TA (2006). A class of membrane proteins shaping the tubular endoplasmic reticulum. Cell.

[56] De Craene J-O (2006). Rtn1p is involved in structuring the cortical endoplasmic reticulum. Mol. Biol. Cell.

[57] Hoffmann PC (2019). Tricalbins contribute to cellular lipid flux and form curved ER-PM contacts that are bridged by rod-shaped structures. Dev. Cell.

[58] Giordano F (2013). PI(4,5)P(2)-dependent and Ca(2+)-regulated ER-PM interactions mediated by the extended synaptotagmins. Cell.

[59] Stephani M (2020). A cross-kingdom conserved ER-phagy receptor maintains endoplasmic reticulum homeostasis during stress. Elife.

[60] Kvam E, Goldfarb DS (2007). Nucleus-vacuole junctions and piecemeal microautophagy of the nucleus in *S. cerevisiae*. Autophagy.

[61] Lips C (2020). Who with whom: functional coordination of E2 enzymes by RING E3 ligases during poly-ubiquitylation. EMBO J..

[62] Ceppi P (2005). Two tail-anchored protein variants, differing in transmembrane domain length and intracellular sorting, interact differently with lipids. Proc. Natl. Acad. Sci..

[63] Pralle A, Keller P, Florin EL, Simons K, Hörber JK (2000). Sphingolipid-cholesterol rafts diffuse as small entities in the plasma membrane of mammalian cells. J. Cell Biol..

[64] Mouyna I (2000). Glycosylphosphatidylinositol-anchored glucanosyltransferases play an active role in the biosynthesis of the fungal cell wall. J. Biol. Chem..

[65] Dolz-Edo L, van der Deen M, Brul S, Smits GJ (2019). Caloric restriction controls stationary phase survival through Protein Kinase A (PKA) and cytosolic pH. Aging Cell.

[66] Aufschnaiter A (2017). The coordinated action of calcineurin and cathepsin D protects against α-synuclein toxicity. Front. Mol. Neurosci..

[67] Pani B (2008). Lipid rafts determine clustering of STIM1 in endoplasmic reticulum-plasma membrane junctions and regulation of store-operated Ca2+ entry (SOCE). J. Biol. Chem..

[68] Pichler H (2001). A subfraction of the yeast endoplasmic reticulum associates with the plasma membrane and has a high capacity to synthesize lipids. Eur. J. Biochem..

[69] Jorgensen JR (2020). ESCRT-III and ER-PM contacts maintain lipid homeostasis. Mol. Biol. Cell.

[70] Babour A, Bicknell AA, Tourtellotte J, Niwa M (2010). A surveillance pathway monitors the fitness of the endoplasmic reticulum to control its inheritance. Cell.

[71] Manford AG, Stefan CJ, Yuan HL, Macgurn JA, Emr SD (2012). ER-to-plasma membrane tethering proteins regulate cell signaling and ER morphology. Dev. Cell.

[72] Mochida K, Nakatogawa H (2020). Atg8-mediated super-assembly of Atg40 induces local ER remodeling in reticulophagy. Autophagy.

[73] Mochida K (2022). Atg39 links and deforms the outer and inner nuclear membranes in selective autophagy of the nucleus. J. Cell Biol..

[74] Insenser M, Nombela C, Molero G, Gil C (2006). Proteomic analysis of detergent-resistant membranes from *Candida albicans*. Proteomics.

[75] Gietz RD, Schiestl RH (2007). High-efficiency yeast transformation using the LiAc/SS carrier DNA/PEG method. Nat. Protoc..

[76] Janke C (2004). A versatile toolbox for PCR-based tagging of yeast genes: New fluorescent proteins, more markers and promoter substitution cassettes. Yeast.

[77] Diessl J, Nandy A, Schug C, Habernig L, Büttner S (2020). Stable and destabilized GFP reporters to monitor calcineurin activity in *Saccharomyces cerevisiae*. Microb. Cell.

[78] Xu J (2019). A fixation method for the optimisation of western blotting. Sci. Rep..

[79] Schindelin J (2012). Fiji: an open-source platform for biological-image analysis. Nat. Methods.

