# Supplementary material for: Partitioning into ER membrane microdomains impacts autophagic protein turnover during cellular aging
Source: Sci Rep. 2024 Jun 13;14:13653. doi: 10.1038/s41598-024-64493-8 (PMC11176346; doi:10.1038/s41598-024-64493-8)
Supplement: Supplementary file 1 — Supplementary Information. [file 41598_2024_64493_MOESM1_ESM.pdf]

Supplementary Material

Partitioning into ER membrane microdomains impacts autophagic protein turnover during cellular aging

Simon Prokisch[1] and Sabrina Büttner[1,#]

[1] Department of Molecular Biosciences, The Wenner-Gren Institute, Stockholm University, 10691 Stockholm, Sweden

# Correspondence:
Sabrina Büttner, sabrina.buettner@su.se

Including:
Supplementary Figures S1-S5
Supplementary Tables S1 and S2

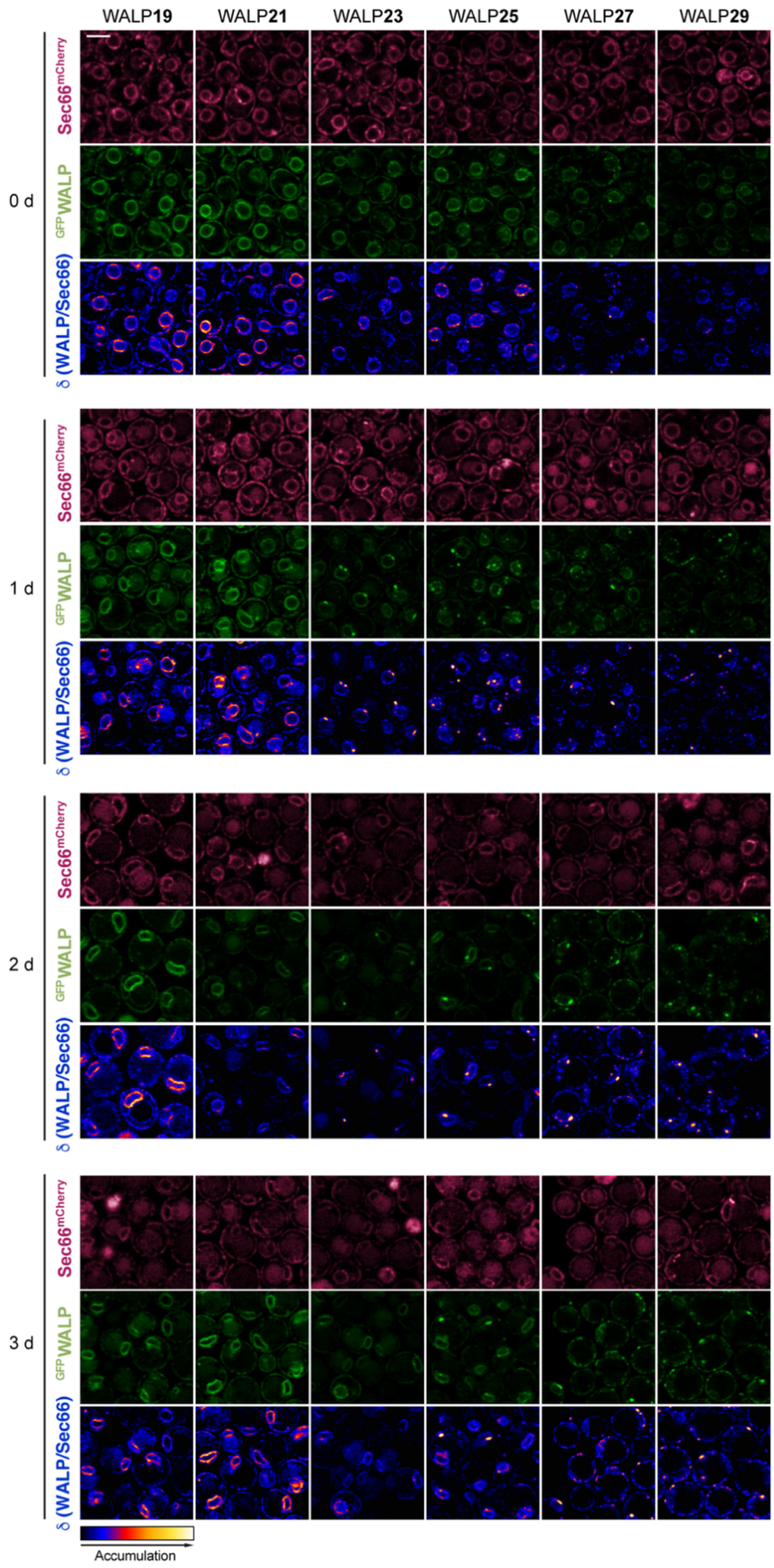

Supplementary Figure S1: Age-dependent reorganization of ER membrane microdomains.

Micrographs of cells endogenously expressing Sec66^{mCherry} as a reference protein and each of the ^{GFP}WALPs to visualize ER membranes thickness in young, growing cells (day 0; corresponding to mid-exponential growth phase) and at different time points during cellular aging. The ratio of ^{GFP}WALP to Sec66^{mCherry} visualizes the ER membrane regions with specific ^{GFP}WALP accumulation. Scale bar: 3 μm.

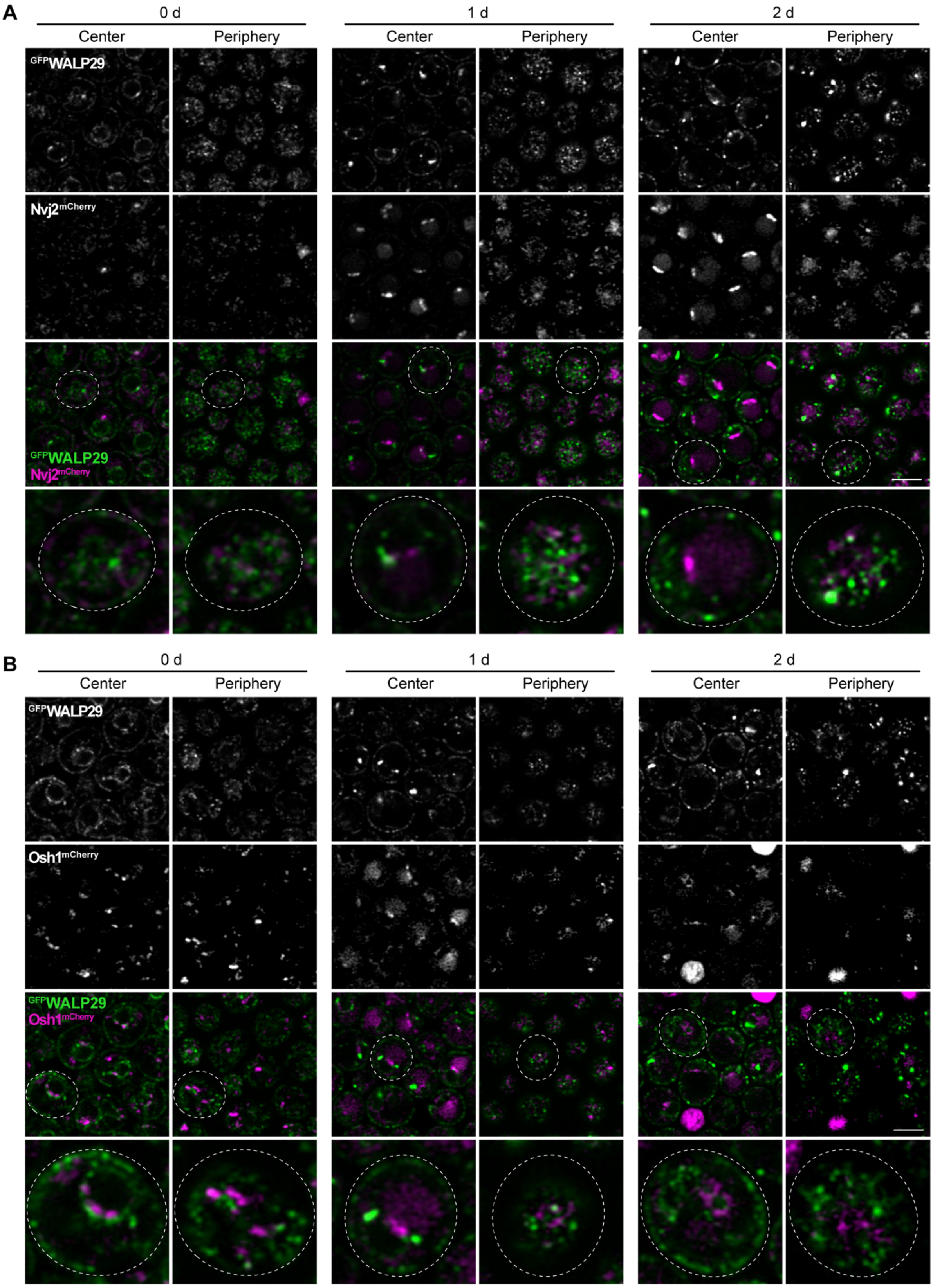

Supplementary Figure S2: Only a small number of WALP29-decorated microdomains form proximal to ER-Golgi contact sites in aging cells.

(A, B) Confocal micrographs of cells endogenously expressing ^{GFP}WALP29 and Nvj2^{mCherry} (A) or Osh1^{mCherry} (B) at indicated time points. A central plane and a peripheral plane of the same cells are shown. Scale bars: 3 μm.

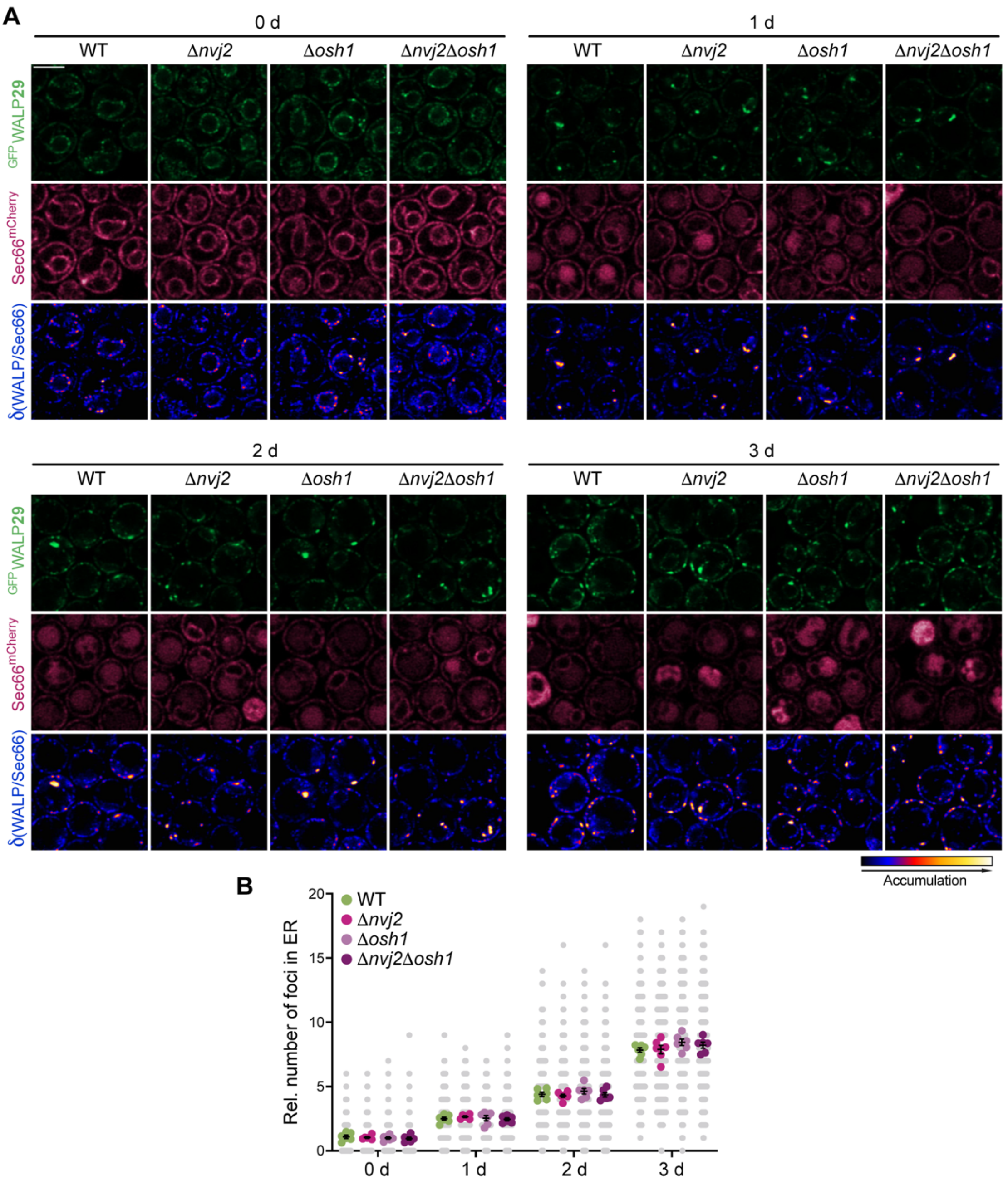

Supplementary Figure S3: Impaired ER-Golgi contact formation via genetic ablation of Nvj2 and Osh1 does not impact the frequency of WALP29-decorated microdomains.

(A) Micrographs of wild type (WT), Δ*nvj2,* Δ*osh1,* and Δ*nvj2*Δ*osh1* cells endogenously expressing Sce66^{mCherry} as a reference protein and ^{GFP}WALP29 to visualize microdomains of increased bilayer thickness in young, growing cells (0 d) and at different time points during cellular aging. Scale bars: 3 μm. **(B)** Quantification of the frequency of WALP29-decorated ER microdomains from confocal micrographs as shown in (A). Gray dots represent individual cells from six independent experiments. Colored dots represent the average for each individual experiment (with 20-46 cells per n), and lines represent the grand mean ± s.e.m. of the individual experiments (n = 6).

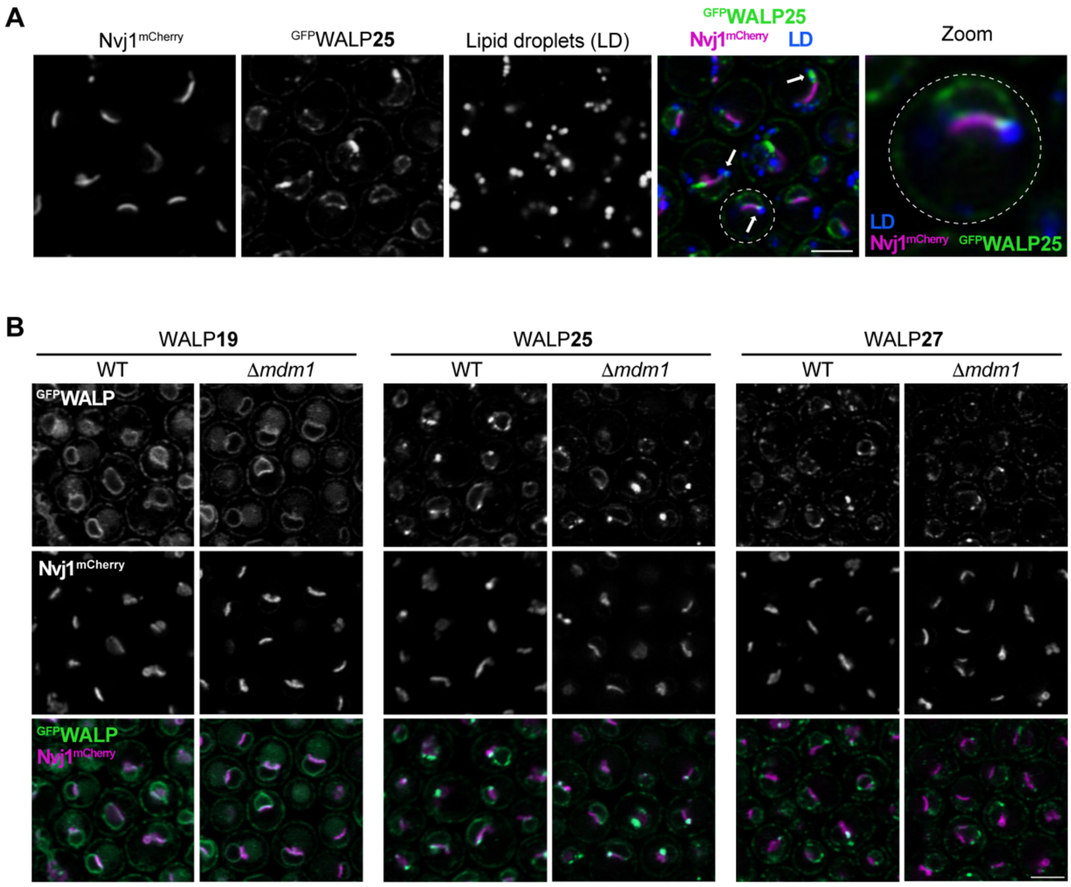

Supplementary Figure S4: Formation of WALP-decorated foci at the rim of the NVJs does not require NVJ-associated lipid droplet clustering.

(A) Micrographs of cells endogenously expressing Nvj1^{mCherry} and ^{GFP}WALP25, stained with monodansylpentane to visualize lipid droplets (LD) after the diauxic shift (day 1). **(B)** Micrographs of wild type (WT) cells and cells lacking the NVJ- and LD-associated protein Mdm1 (Δ*mdm1*), endogenously expressing Nvj1^{mCherry} and ^{GFP}WALP19, ^{GFP}WALP25 or ^{GFP}WALP27, after the diauxic shift (day 1). Scale bars: 3 μm.

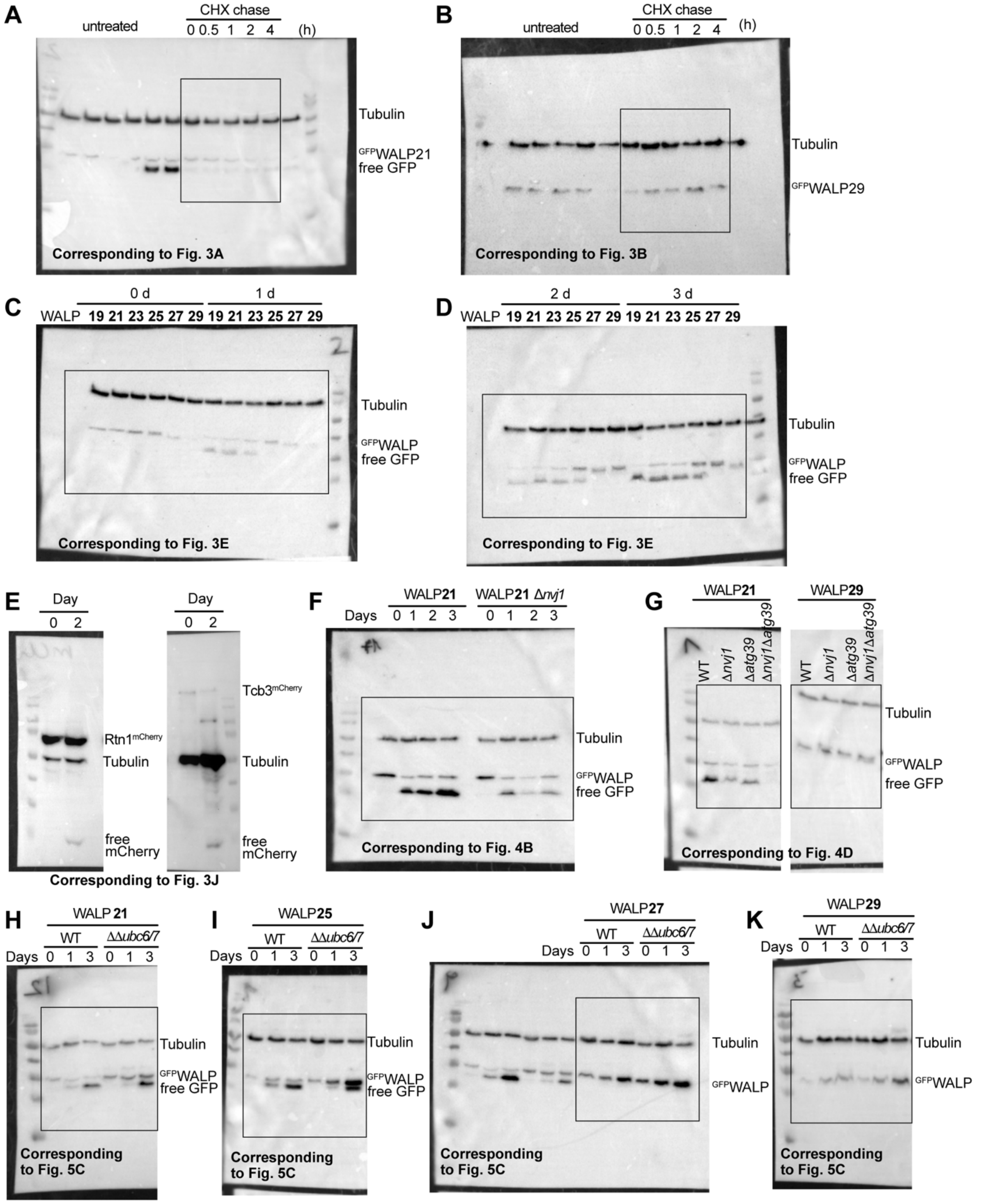

Supplementary Figure S5: Full length immunoblots shown in this study.

(A-K) Unprocessed, full length immunoblots corresponding to the cropped immunoblots shown in Figure 3 (A-E), in Figure 4 (F, G) and Figure 5 (H-K). Blots have been probed with antibodies directed against the GFP-epitope, the mCherry-epitope and tubulin as loading control as indicated.

Supplementary Table S1: Yeast strains used in this study.

Strain	Genotype	Source
WT (BY4741)	MAT**a**, *his3*Δ1, *leu2*Δ0, *met15*Δ0, *ura3*Δ0	Euroscarf
WT ^{GFP}WALP19	BY4741 *GFP-WALP19::His3MX*	Prasad *et al.*, 2020
WT ^{GFP}WALP21	BY4741 *GFP-WALP21::His3MX*	Prasad *et al.*, 2020
WT ^{GFP}WALP23	BY4741 *GFP-WALP23::His3MX*	Prasad *et al.*, 2020
WT ^{GFP}WALP25	BY4741 *GFP-WALP25::His3MX*	Prasad *et al.*, 2020
WT ^{GFP}WALP27	BY4741 *GFP-WALP27::His3MX*	Prasad *et al.*, 2020
WT ^{GFP}WALP29	BY4741 *GFP-WALP29::His3MX*	Prasad *et al.*, 2020
WT ^{GFP}WALP19 Sec66^{mCherry}	BY4741 *GFP-WALP19::His3MX, SEC66-mCherry::kanMX*	This study
WT ^{GFP}WALP21 Sec66^{mCherry}	BY4741 *GFP-WALP21::His3MX, SEC66-mCherry::kanMX*	This study
WT ^{GFP}WALP23 Sec66^{mCherry}	BY4741 *GFP-WALP23::His3MX, SEC66-mCherry::kanMX*	This study
WT ^{GFP}WALP25 Sec66^{mCherry}	BY4741 *GFP-WALP25::His3MX, SEC66-mCherry::kanMX*	This study
WT ^{GFP}WALP27 Sec66^{mCherry}	BY4741 *GFP-WALP27::His3MX, SEC66-mCherry::kanMX*	This study
WT ^{GFP}WALP29 Sec66^{mCherry}	BY4741 *GFP-WALP29::His3MX, SEC66-mCherry::kanMX*	This study
WT ^{GFP}WALP19 Nvj1^{mCherry}	BY4741 *GFP-WALP19::His3MX, NVJ1-mCherry::kanMX*	This study
WT ^{GFP}WALP21 Nvj1^{mCherry}	BY4741 *GFP-WALP21::His3MX, NVJ1-mCherry::kanMX*	This study
WT ^{GFP}WALP23 Nvj1^{mCherry}	BY4741 *GFP-WALP23::His3MX, NVJ1-mCherry::kanMX*	This study
WT ^{GFP}WALP25 Nvj1^{mCherry}	BY4741 *GFP-WALP25::His3MX, NVJ1-mCherry::kanMX*	This study
WT ^{GFP}WALP27 Nvj1^{mCherry}	BY4741 *GFP-WALP27::His3MX, NVJ1-mCherry::kanMX*	This study
WT ^{GFP}WALP29 Nvj1^{mCherry}	BY4741 *GFP-WALP29::His3MX, NVJ1-mCherry::kanMX*	This study
Δ*nvj1* ^{GFP}WALP21	BY4741 *GFP-WALP21::His3MX, nvj1*Δ*::hphNT1*	This study
Δ*nvj1* ^{GFP}WALP29	BY4741 *GFP-WALP29::His3MX, nvj1*Δ*::hphNT1*	This study
Δ*nvj1*Δ*atg39* ^{GFP}WALP21	BY4741 *GFP-WALP21::His3MX, nvj1*Δ*::hphNT1, atg39*Δ*::kanMX*	This study
Δ*nvj1*Δ*atg39* ^{GFP}WALP29	BY4741 *GFP-WALP29::His3MX, nvj1*Δ*::hphNT1, atg39*Δ*::kanMX*	This study
Δ*atg39* ^{GFP}WALP21	BY4741 *GFP-WALP21::His3MX, atg39*Δ*::kanMX*	This study
Δ*atg39* ^{GFP}WALP29	BY4741 *GFP-WALP29::His3MX, atg39*Δ*::kanMX*	This study
$\Delta\Delta$*ubc6/7* ^{GFP}WALP21	BY4741 *GFP-WALP21::His3MX, ubc6*Δ*::hphNT1, ubc7*Δ*::kanMX*	This study
$\Delta\Delta$*ubc6/7* ^{GFP}WALP25	BY4741 *GFP-WALP25::His3MX, ubc6*Δ*::hphNT1, ubc7*Δ*::kanMX*	This study
$\Delta\Delta$*ubc6/7* ^{GFP}WALP27	BY4741 *GFP-WALP27::His3MX, ubc6*Δ*::hphNT1, ubc7*Δ*::kanMX*	This study
$\Delta\Delta$*ubc6/7* ^{GFP}WALP29	BY4741 *GFP-WALP29::His3MX, ubc6*Δ*::hphNT1, ubc7*Δ*::kanMX*	This study
WT ^{GFP}WALP29 Nvj2^{mCherry}	BY4741 *GFP-WALP29::His3MX, NVJ2-mCherry::kanMX*	This study
WT ^{GFP}WALP29 Osh1^{mCherry}	BY4741 *GFP-WALP29::His3MX, OSH1-mCherry::kanMX*	This study
WT ^{GFP}WALP29 Rtn1^{mCherry}	BY4741 *GFP-WALP29::His3MX, RTN1-mCherry::kanMX*	This study
WT ^{GFP}WALP29 Tcb3^{mCherry}	BY4741 *GFP-WALP29::His3MX, TCB3-mCherry::kanMX*	This study
Δ*mdm1* ^{GFP}WALP19	BY4741 *GFP-WALP19::His3MX, mdm1*Δ*:: hphNT1*	This study
Δ*mdm1* ^{GFP}WALP25	BY4741 *GFP-WALP25::His3MX, mdm1*Δ*:: hphNT1*	This study
Δ*mdm1* ^{GFP}WALP27	BY4741 *GFP-WALP27::His3MX, mdm1*Δ*:: hphNT1*	This study
Δ*osh1* ^{GFP}WALP29 Sec66^{mCherry}	BY4741 *GFP-WALP29::His3MX, osh1*Δ*:: LEU2, SEC66-mCherry::kanMX*	This study
Δ*nvj2* ^{GFP}WALP29 Sec66^{mCherry}	BY4741 *GFP-WALP29::His3MX, nvj2*Δ*:: natNT2, SEC66-mCherry::kanMX*	This study
Δ*nvj2*Δ*osh1* ^{GFP}WALP29 Sec66^{mCherry}	BY4741 *GFP-WALP29::His3MX, nvj2*Δ*:: natNT2, osh1*Δ*:: LEU2, SEC66-mCherry::kanMX*	This study

Supplementary Table S2: Oligonucleotides used in this study.

Modification	Primer	Oligonucleotide (5'-3')	Template
Tagging of *SEC66*	Sec66 S2	AACACTGAACGAGCGAATACATATCTTTGCACACAGTAGGCACTAATCGATGAATTCGAGCTCG	pSB89; pYM-C (kanMX) mCherry linker from pYM25 (Janke *et al*., 2004)
	Sec66 S3	AAAGAGTGGGAGCTGAAAATAAATAATGATGGAAGATTAGTCAATCGTACGCTGCAGGTCGAC	
	Control	AGTGACGTTATGGATGGTAG	
Tagging of *NVJ1*	Nvj1 S2	CTCGTTGTAAGTGACGATGATAACCGAGATGACGGAAATATAGTACATTAATCGATGAATTCGAGCTCG	pSB89; pYM-C (kanMX) mCherry linker from pYM25 (Janke *et al*., 2004)
	Nvj1 S3	CTAGATGCACAAGTGAACACTGAACAAGCATACTCTCAACCATTTAGATACCGACGCTGCAGGTCGAC	
	Control	CTATTGACCACATAATCCTTAG	
Tagging of *NVJ2*	Nvj2 S2	GCATATAGCTTCAAGTGATATTTATTTATTTTTAATATAGTACCGTGGACTCAATCGATGAATTCGAGCTCG	pSB89; pYM-C (kanMX) mCherry linker from pYM25 (Janke *et al*., 2004)
	Nvj2 S3	CGGCTTTTTCAAGCAAGATTTAGAATTTGAAGAACAGCGAGAGCCCAAACTGCGTACGCTGCAGGTCGAC	
	Control	ATATTCACACTGTACTAGAT	
Tagging of *OSH1*	Osh1 S2	AATGGATACAAATGAACGAGTGTTATTGTGACTACATTGCACAGCTTAATCGATGAATTCGAGCTCG	pSB89; pYM-C (kanMX) mCherry linker from pYM25 (Janke *et al*., 2004)
	Osh1 S3	GTATTGGAACAAAAGAAAAAATCATGACTTTAAAGATTGTGCTGATATTTTCCGTACGCTGCAGGTCGAC	
	Control	GTATCGGCTTTGAATCCATG	
Tagging of *TCB3*	Tcb3 S2	CACACCAAATGTGCCCTTATTGAGCGTATAAAAGAATAGTTTTCACTGTTTATTAATCGATGAATTCGAGCTCG	pSB89; pYM-C (kanMX) mCherry linker from pYM25 (Janke *et al*., 2004)
	Tcb3 S3	CAAGAATGGTCAGGTACCTCCCGTGCCAGAAGTTCCTCAAGAATACACGCAGCGTACGCTGCAGGTCGAC	
	Control	CCACTGGTGGTTTAAAGAAAG	
Tagging of *RTN1*	Rtn1 S2	GAGACAAAAGTTAGCTATTCTTGTTTGAAATGAAAAAAAAAAAGCACTCAATCGATGAATTCGAGCTCG	pSB89; pYM-C (kanMX) mCherry linker from pYM25 (Janke *et al*., 2004)
	Rtn1 S3	GAAGAAAAGTACAAAAAACTTGCAAAATGAATTGGAAAAAAACAACGCTCGTACGCTGCAGGTCGAC	
	Control	CAGTCATCTCGAGCAAAATCCC	
Deletion of *NVJ1*	Nvj1 S1	TGTGCATAATATCAAAAAAGCTACAAATATAATTGTAAAATATAATAAGCATGCGTACGCTGCAGGTCGAC	pFA6a-hphNT1 (Janke *et al*., 2004)
	Nvj1 S2	CTCGTTGTAAGTGACGATGATAACCGAGATGACGGAAATATAGTACATTAATCGATGAATTCGAGCTCG	
	Control	CTATTGACCACATAATCCTTAG	

	Atg39 S1	GCAGTGACGATAATAGAGACTAGTAAAACAGTCGAGT TGTCGGACCTAAAATGCGTACGCTGCAGGTCGAC	
Deletion of *ATG39*	Atg39 S2	CGTTTTTTTTTTCTTTTGTTAATTTCATTCTTCATGCTGG GTTTTGGATGATCTAATCGATGAATTCGAGCTCG	pFA6a-kanMX (Bähler *et al.*, 1998)
	Control	GCTGCATATTTGCTTTCGCCG	
	Ubc6 S1	ACCGCATTCGCAAATTGCAAACAAAGTACGTACAATA GTAATGCGTACGCTGCAGGTCGAC	
Deletion of *UBC6*	Ubc6 S2	GTGTTGTCAAAATTTATCTAAAGTTTAGTTCATTTAATG GCTTCAATCGATGAATTCGAGCTCG	pFA6a-hphNT1 (Janke *et al.*, 2004)
	Control	ACCCTAGCGCCAATGCAAGA	
	Ubc7 S1	GGAACTTCCCTAGTAATAGTGTAATTTGGAAGGGCAT AGCATGCGTACGCTGCAGGTCGAC	
Deletion of *UBC7*	Ubc7 S2	GTTAAAAGGAAGACCAAATGATCATTAACCTGCTACCT GCTTTCAATCGATGAATTCGAGCTCG	pFA6a-kanMX (Bähler *et al.*, 1998)
	Control	CCAAAGATTTCCATAATGAT	
	Nvj2 S1	ACACATCGAAGAGCAGAACAGCAAGAGAAAAGTAGC ATTAAAAGACCATAATGCGTACGCTGCAGGTCGAC	
Deletion of *NVJ2*	Nvj2 S2	GCATATAGCTTCAAGTGATATTTATTTATTTTTAATATA GTACCGTGGACTCAATCGATGAATTCGAGCTCG	pFA6a-natNT2 (Janke *et al.*, 2004)
	Control	GTTCAATGTAGGTAATGATG	
	Mdm1 S1	GAAAGCGCCATAAGTGCGCGTGTTTGTGCCTTCTGATA TGATATCGTATGCGTACGCTGCAGGTCGAC	
Deletion of *MDM1*	Mdm1 S2	CAATTACACTTTTTTTTTTAGATTGTTCGGTACTTAGTC AAGTTTTATTTTCAATCGATGAATTCGAGCTCG	pFA6a-hphNT1 (Janke *et al.*, 2004)
	Control	CGTCAAGGGTATCAGCAGAG	
	Osh1 S1	TAAAAAGGGAAAAGTTTAAACATCAAAGTACACCTTTC ACCCCTCCACACACCATGCGTACGCTGCAGGTCGAC	
Deletion of *OSH1*	Osh1 S2	AATGGATACAAATGAACGAGTGTTATTGTGACTACATT GCACAGCTTAATCGATGAATTCGAGCTCG	pFA6a-LEU2 (this study)
	Control	GTATCGGCTTTGAATCCATG	

References

Bähler, J. *et al*. Heterologous modules for efficient and versatile PCR-based gene targeting in Schizosaccharomyces pombe. *Yeast* **10**, 943-51 (1998)

Janke, C. *et al*. A versatile toolbox for PCR-based tagging of yeast genes: new fluorescent proteins, more markers and promoter substitution cassettes. *Yeast* **21**, 947–962 (2004).

Prasad, R., Sliwa-Gonzalez, A. & Barral, Y. Mapping bilayer thickness in the ER membrane. *Sci Adv* **6**, eaba5130 (2020).
